# Flavonoids: Potential New Drug Candidates for Attenuating Vascular Remodeling in Pulmonary Hypertension

**DOI:** 10.3390/ijms27010210

**Published:** 2025-12-24

**Authors:** Xiaoyi Zhang, Mingshu Chen, Ranran Wang, Ruiqi Liu, Difei Gong, Meng Zhang, Yangyang He, Guanhua Du, Lianhua Fang, Tianyi Yuan

**Affiliations:** 1 Beijing Key Laboratory of Innovative Drug Discovery and Polymorphic Research for Cerebrovascular Diseases, Institute of Materia Medica, Chinese Academy of Medical Sciences and Peking Union Medical College, Beijing 100050, China; zxybucm@aliyun.com (X.Z.); chenmingshu@imm.ac.cn (M.C.); wrrpumc@163.com (R.W.); liuruiqi@imm.ac.cn (R.L.); gongdf1@126.com (D.G.); dugh@imm.ac.cn (G.D.); 2 State Key Laboratory of Bioactive Substances and Functions of Natural Medicines, Institute of Materia Medica, Chinese Academy of Medical Sciences and Peking Union Medical College, Beijing 100050, China; 3 School of Pharmacy, Henan University, Kaifeng 475004, China; zhangmzmm@163.com (M.Z.); heyangyang@vip.henu.edu.cn (Y.H.)

**Keywords:** flavonoids, pulmonary arterial remodeling, pulmonary hypertension, antioxidant, anti-inflammation

## Abstract

Pulmonary hypertension (PH) is a progressive and life-threatening disorder characterized by elevated pulmonary arterial pressure, leading to right ventricular remodeling and significant mortality. Pulmonary arterial remodeling, a critical pathological feature of PH, refers to structural alterations in the pulmonary vasculature driven by various pathogenic factors. Targeting this remodeling process has emerged as a promising strategy for treating and potentially curing the disease. In recent years, growing interest has been directed toward exploring natural products as anti-PH agents. Among them, flavonoids have demonstrated potent efficacy in the cardiopulmonary system. As a prominent class of natural small-molecule compounds, flavonoids exhibit broad biological activities, such as antioxidant, anti-inflammatory, and anti-proliferative properties. They have shown the ability to inhibit remodeling and restore vascular function across various vessel types, including pulmonary arteries. This review summarizes the effects of flavonoids on PH, with emphasis on their inhibition of pulmonary arterial remodeling. We also discuss the therapeutic potential of flavonoids in PH and discuss their underlying mechanisms of action. These insights may guide the development of next-generation PH therapeutics, either through the utilization of flavonoid-based structures or the preparation of compound formulations containing flavonoids.

## 1. Introduction

Pulmonary hypertension (PH) is a life-threatening syndrome characterized by pulmonary arterial remodeling and increased resistance, which leads to right ventricular remodeling and heart failure with high mortality [1]. PH is classified into five groups. Although PH overall is not a rare disease, specific types qualify as rare diseases, with reported prevalence as low as 5–10 cases per million [2]. A critical evolution in PH has been the redefinition of its hemodynamic criteria. Initially set at mean pulmonary arterial pressure (mPAP) ≥ 25 mmHg at the first World Symposia on Pulmonary Hypertension (WSPH), the diagnostic threshold was later recommended to be reduced to mPAP > 20 mmHg during the 6th WSPH [3]. In the 2022 European Society of Cardiology/European Respiratory Society guideline, this suggestion was formally incorporated [4]. This change in diagnostic criteria potentially indicates a significant increase in the number of PH patients in the near future, consequently accelerating the demand for effective PH pharmacotherapies.

The current pharmacological management of PH involves two primary drug classes. One class includes general agents, such as diuretics, anticoagulants, and vasodilators. The other class encompasses targeted therapies that act on specific pathways implicated in PH, including endothelin receptor antagonists, phosphodiesterase type 5 (PDE5) inhibitors, soluble guanylate cyclase (sGC) activators, and prostacyclin-based analogs and receptor agonists [5]. In 2024, the first-in-class drug sotatercept, an activin receptor IIA-Fc fusion protein, was approved, representing a major breakthrough in the therapeutic field.

Although targeted pharmacological therapies have extended patient survival in PH, they predominantly ameliorate vascular dysfunction without substantially reversing pulmonary arterial remodeling, failing to provide a fundamental cure for the disease. This critical shortcoming has motivated increased research into anti-PH agents derived from natural products, many of which exhibit multi-target mechanisms and pronounced therapeutic effects on pulmonary arterial remodeling.

In the present review, we aim to summarize the effects of flavonoids, a prominent class of natural small-molecule compounds, on PH, with a particular focus on their attenuation of pulmonary arterial remodeling. We discuss and analyze the potential of flavonoids as PH therapeutics by elucidating their anti-remodeling efficacy and the underlying mechanisms, thereby outlining novel avenues for future drug development centered on this compound class.

### 1.1. Pulmonary Arterial Remodeling Is the Key Pathological Factor for PH

**Figure 1:**
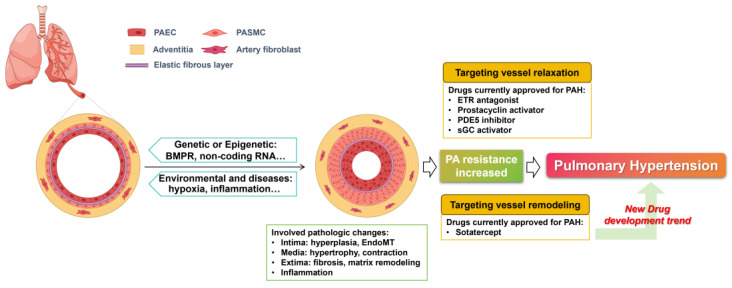
Vascular remodeling is the key factor for the occurrence and development of PH, the inhibition of pulmonary arterial remodeling is the trend of drug discovery and development in the future.

Vascular remodeling refers to changes in vascular structure caused by injury factors such as hypoxia, inflammation, and high-blood-flow shear stress. It represents a critical pathological feature and a key driver of numerous cardiovascular diseases [6]. In PH, the remodeling of pulmonary arteries (PAs) is the most prominent pathological manifestation, which mainly involves the distal pulmonary arterioles characterized by inflammatory intimal hyperplasia, endothelial–mesenchymal transition (EndoMT), media hypertrophy with sustained contraction, as well as adventitia fibrosis and extracellular matrix remodeling [7]. As pulmonary arterial remodeling is established as the central pathogenic mechanism in PH, deciphering its intricate processes has become a major research focus, driving efforts to elucidate underlying mechanisms and discover novel targeted drugs (Figure 1).

Pulmonary arterial remodeling arises from a complex interplay of genetic, epigenetic, and environmental factors. Genetically, mutations in bone morphogenetic protein receptor type 2 (BMPR2), which is a crucial protective gene in endothelial and smooth muscle cells, have become the predominant genetic lesion in heritable PH [8]. Epigenetically, factors such as non-coding RNAs and modifying enzymes actively regulate the remodeling process [9,10]. Concurrently, environmental triggers and primary diseases (e.g., hypoxia, toxins, lung and autoimmune diseases) significantly contribute to pulmonary arterial remodeling. These factors can lead to DNA damage, initiating repair responses that promote apoptosis resistance and hyperproliferation, which are key events in vascular remodeling [11]. Irrespective of the initiating cause, pulmonary arterial remodeling is a core pathological driver of PH progression. This understanding has shifted the therapeutic paradigm, establishing that effective treatment must combine vasodilation with direct anti-remodeling strategies.

Despite the central role of remodeling, existing PH drugs primarily focus on vasorelaxation, leaving a critical therapeutic gap. The recent approval of sotatercept (WINREVAIR™), a novel fusion protein that rebalances pro-proliferative signaling by trapping activins and growth and differentiation factors, marks a turning point [12]. Its proven efficacy in reversing pulmonary arterial and right ventricle remodeling [13,14] validates the therapeutic potential of targeting remodeling pathways. The success of sotatercept poses a pivotal question of how to discover novel drug candidates capable of mitigating pulmonary arterial remodeling for PH treatment. Natural products and their derivative components represent a promising and rich source for such discoveries.

### 1.2. Flavonoids Compounds Have Beneficial Effects on PH

Natural products, including those found in traditional Chinese medicine, represent a valuable resource for novel drug discovery. Among the numerous small natural molecules, flavonoids have gained significant research interest. As a class of natural compounds, flavonoids are widely distributed in plants as secondary metabolites, commonly found in fruits, vegetables, tea, and other botanicals [15].

**Figure 2:**
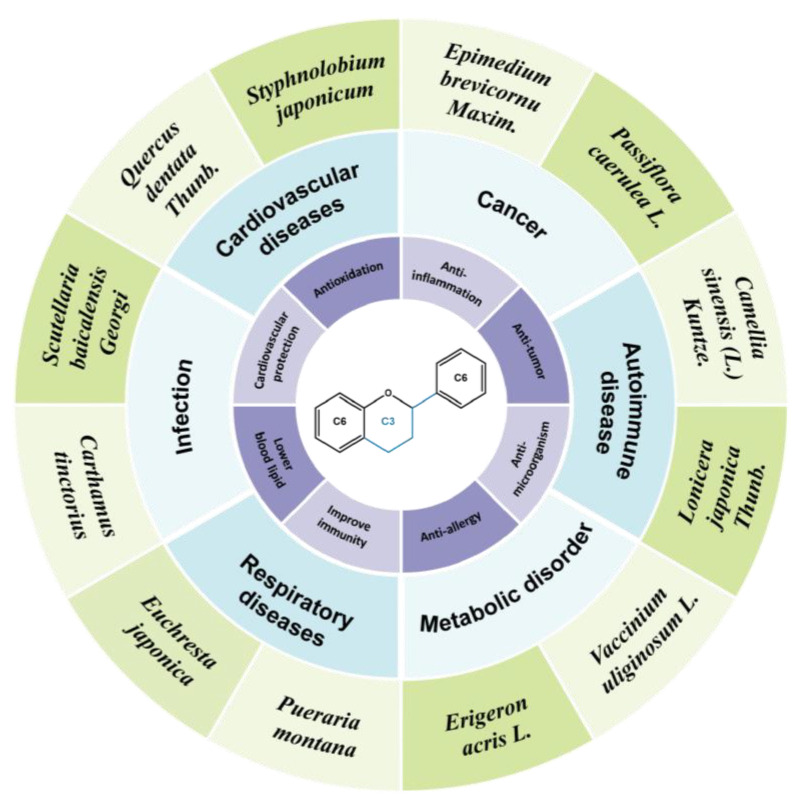
The structural skeleton of flavonoids and an overview of flavonoids: the pharmacology bioactivities and plant sources.

Structurally, flavonoids were traditionally regarded as derivatives of 2-phenylchromone. The concept has since been expanded to include compounds featuring two benzene rings (A ring and B ring) connected by a C3 chain, forming a characteristic C6–C3–C6 skeleton (Figure 2). Although the flavonoid family now encompasses many structural variants, this review focuses primarily on those conforming to the C6–C3–C6 skeleton. Based on the B-ring linkage site, oxidation state of the central three-carbon chain, and its cyclization pattern, these flavonoids are systematically classified into flavones, flavanols, dihydroflavones, dihydroflavonols, isoflavones, isoflavonols, chalcones, aurones, and anthocyanidins (Table 1). Despite their structural diversity, the core scaffold of flavonoids remains relatively simple. In plants, most flavonoids occur as glycosides, with only a minor fraction present in free form.

Flavonoids demonstrate a broad spectrum of pharmacological activities, including cholesterol- and glucose-lowering, antibacterial, antiviral, anticancer activity, as well as antioxidant and anti-inflammatory abilities [16,17]. Among these, their antioxidant and anti-inflammatory properties are particularly noteworthy. Extensive research has demonstrated the cardiovascular benefits of flavonoids, with some already in clinical use. For instance, *Puerarin* injection and total flavonoids from *Ginkgo biloba* L. have been shown to dilate coronary arteries and improve vascular smooth muscle function, making them effective clinical treatments for coronary heart disease [18,19].

Numerous studies support the therapeutic potential application of flavonoids in targeting vascular remodeling and treating PH (Table 2). Furthermore, specific flavonoids have been demonstrated to inhibit vascular remodeling, yet their effects on PH have not been specifically documented in the literature (Table 3). Up to November 2025, the international literature includes about 210 English-language papers on the anti-PH efficacy of flavonoids and another 246 on their anti-vascular remodeling effects, in contrast to over 2000 Chinese papers on relevant topics. This review briefly summarized international reports on anti-PH flavonoids, with a particular focus on analyzing their roles and regulatory effects across various pathological mechanisms of vascular remodeling.

## 2. Pathological Mechanisms Underlying Pulmonary Arterial Remodeling

The structure and function of the PAs are characteristic. In contrast to systemic arteries, PAs transport deoxygenated blood, which contributes to the PAs’ distinctive response to hypoxic conditions. PAs exhibit vasoconstriction in low oxygen conditions, which is named hypoxic pulmonary vasoconstriction (HPV). While HPV is an adaptive mechanism for optimizing pulmonary gas exchange [95], prolonged hypoxia or lung pathologies can induce a shift from this physiological response to a pathological state. In this state, pulmonary arterial endothelial and smooth muscle cells undergo phenotypic transformation and hyperproliferation, contributing to the pathological vascular remodeling and increased resistance. Beyond hypoxia, other critical contributors to this remodeling process include inflammation, oxidative stress, genetic factors, and certain primary diseases.

**Figure 3:**
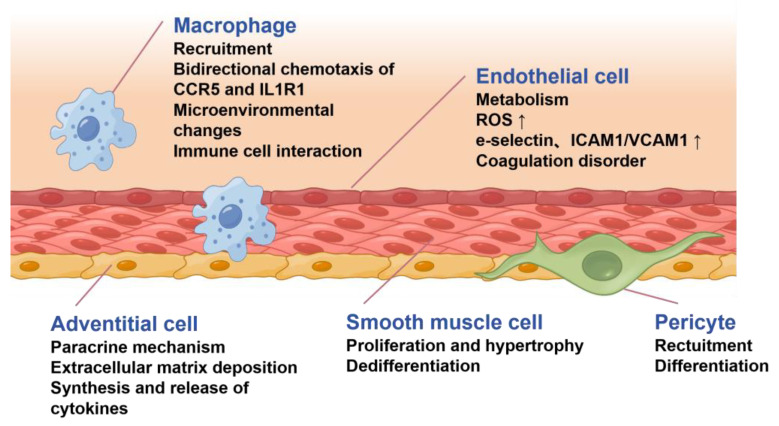
Pathological changes underlying pulmonary arterial remodeling involve phenotype transformation and dysfunction in multiple kinds of cells.

During the development of PH, the remodeling of PAs involves nearly all types of cells from the intima, media, and adventitia (Figure 3). The endothelium plays a dominant role in the regulation of PA function. Its injury or dysfunction is considered an initial cause in remodeling. The muscle layer of the PA is structurally thinner and operates under much lower pressure (typically < 20 mmHg) than systemic arteries, accounting for the inefficacy of most antihypertensive drugs in treating PH. Following endothelial insult, the phenotypic transformation of PASMCs becomes a key driver of remodeling, leading to medial thickening. And the adventitia further amplifies this process through the release of various profibrotic and pro-proliferative factors.

### 2.1. Endothelial Dysfunction and Endothelial–Mesenchymal Transition

The vascular endothelium plays an important role in maintaining cardiovascular homeostasis [96]. As the inner lining of the vascular wall, endothelial cells are subject to the fluid shear stress generated by blood flow. The mechanical stimulus regulates endothelial cell structure and function, thereby influencing key vascular physiological processes, including vascular remodeling and angiosthenia regulation [97]. Physiologically, fluid shear stress serves as the primary stimulus for the sustained production of NO, a vasodilator that represents a central therapeutic target in antihypertensive drug development [98,99]. In PH, therapeutics targeting the NO pathway demonstrate potent efficacy, such as PDE5 inhibitors [100] and inhaled NO [101]. Another critical endothelial-derived vasoactive substance, ET, also plays a major pathogenic role in PH, and endothelin receptor antagonists are currently established as first-line therapeutic agents in clinical practice [102].

The imbalance between NO and ET-1 drives endothelial dysfunction by promoting the expression of adhesion molecules, aberrant local release of chemokines, cytokines, and growth factors, as well as increased reactive oxygen species (ROS) [103,104]. This disrupted balance is a hallmark of PH-associated endothelial pathology and accelerates the progression of the disease.

Endothelial dysfunction also promotes a pro-thrombotic state and contributes to the formation of in situ thrombosis. Caveolin-1, an endothelial structural protein, participates in the production of NO [105]. NO normally prevents platelet adhesion to the vessel wall and exerts antithrombotic effects; therefore, reduced NO resulting from endothelial dysfunction increases platelet deposition and elevates the risk of thrombus formation in the pulmonary vasculature [106].

EndoMT further contributes to pulmonary arterial remodeling by transforming endothelial cells into smooth muscle-like cells with enhanced proliferation and migration capacity [6]. Recognized as an important pathological character and a potential therapeutic target in PH, EndoMT involves the loss of characteristic endothelial cobblestone morphology and typical markers, such as platelet endothelial cell adhesion molecule-1 (CD31), Vascular endothelial cadherin (VE-cadherin), and vascular endothelial calreticulin, and acquires a mesenchymal phenotype expressing epithelial–mesenchymal transition marker vimentin and myofibroblast marker α-smooth muscle actin (α-SMA) [107]. This transition is accompanied by disruption of intercellular junctions, enabling endothelial cells to gain the ability to migrate and invade, detach from the tightly adherent monolayer of the lumen, and migrate toward the internal tissue [108].

Beyond endothelial cells, pericytes, a subpopulation of perivascular cells located at the interface between the endothelium and surrounding tissue, also play an important role in PH endothelial remodeling. Single-cell RNA sequencing has identified two distinct *Higd1b*^+^ pericyte subpopulations in pulmonary capillaries. Among these, type-2 pericytes localize around small arteries. Under hypoxic conditions, they rapidly proliferate, upregulate smooth muscle markers (e.g., vimentin and transgelin), and switch to a myogenic phenotype that directly promotes distal pulmonary arterial remodeling [109].

### 2.2. Smooth Muscle Cell Phenotypic Transformation

Vascular smooth muscle cells are highly specialized cells that have two phenotypes: contractile phenotype and synthetic phenotype. Local smooth muscle cell transformation is the main source of medial cell remodeling. Under normal circumstances, mature smooth muscle cells eventually differentiate into a contractile phenotype. Under pathological conditions, smooth muscle cells undergo dedifferentiation, transforming from a contractile to a synthetic phenotype that confers strong capacities for proliferation and migration ability [110].

The interplay between ROS and inflammation acts synergistically to the phenotypic switch. Inflammatory cytokines such as TNFα and interleukin-1β (IL-1β) induce a rapid generation of ROS. The ensuing ROS then suppresses α-SMA while up-regulating vimentin and osteopontin, thereby driving the transition toward the synthetic phenotype [111].

### 2.3. Vascular Adventitia Remodeling

As the outermost layer of the vessel wall, the vascular adventitia is a complex tissue. Its key cellular constituents are fibroblasts, but it also contains immune cells, progenitor cells, nerve fibers, and lymphatic vessels. Despite being frequently overlooked in the pathological remodeling of pulmonary hypertensive vessels, its thickening represents the earliest and most prominent change in PH [112]. Adventitial fibrosis is associated with luminal narrowing and compromises the vasodilatory responsiveness of the vessel wall. Hypoxia further upregulates carbonic anhydrase activity in the adventitia, exacerbating vascular inflammation and remodeling [113].

### 2.4. Other Mechanisms Underlying Pulmonary Arterial Remodeling

The pathogenesis of PH is multifactorial, involving a network of complex pathophysiological mechanisms. Aside from those previously detailed, a range of other processes are involved in the remodeling of PAs. For instance, enhanced collagen accumulation and cross-linking in the extracellular matrix (ECM), together with elevated tenascin and fibronectin, collectively increase vascular stiffness, thereby inducing significant ECM alterations. Other contributing factors encompass abnormal humoral control, disrupted cell adhesion, and the formation of vascular shunts [114]. Consequently, a therapeutic approach that simultaneously addresses the multiple pathways implicated in pulmonary arterial remodeling holds considerable promise for the treatment of PH.

## 3. Flavonoids Suppress Oxidative Stress and Inflammation in Pulmonary Arterial Remodeling

### 3.1. Flavonoids Protects Pulmonary Arteries from Oxidative Stress Injury

Oxidative stress results from a disruption in the equilibrium between free radicals and antioxidants, which leads to cellular damage. This condition is characterized by an overabundance of oxidants, including ROS and reactive nitrogen species, that exceed the body’s antioxidant capacity during pathogenesis [115]. Surplus ROS directly or indirectly inflicts damage on cellular proteins, lipids, and DNA, triggering protein denaturation, lipid peroxidation, and gene mutation. These alterations can promote significant cellular impairment and inflammatory responses [116].

In the pathological mechanisms of PH, hypoxia contributes to pathological vascular remodeling through inducing oxidative stress. This can trigger sustained contraction of the PA, leading to vascular dysfunction and remodeling [117]. Hypoxia also upregulates nicotinamide adenine dinucleotide phosphate (NADPH) oxidase expression in both PAECs and PASMCs and stimulates the conversion of xanthine dehydrogenase to xanthine oxidase in endothelial cells, collectively elevating superoxide levels in lung tissue [118].

**Figure 4:**
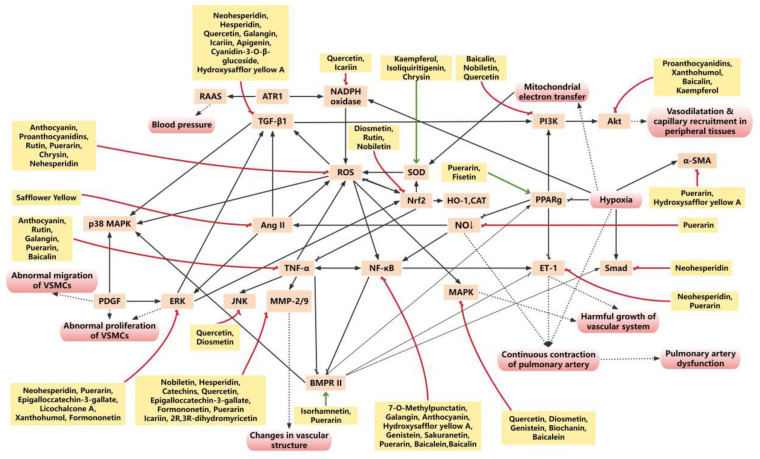
The main molecular mechanism network related to oxidative stress of flavonoids in PH vascular remodeling. Various flavonoids regulate the process of vascular remodeling through multiple effects, multiple targets and multiple pathways. Red box, biological effects; orange box, target; yellow box, flavonoids; red arrow, inhibition; green arrows, promotion.

Flavonoids are recognized for their broad pharmacological profile, with a notable antioxidant capability. Extensive evidence indicates that flavonoids inhibit ROS generation and suppress oxidative stress through multiple targets (Figure 4).

*Puerarin*, a major isoflavone glycoside extracted from *Pueraria lobata*, exhibits a wide range of biological activities with therapeutic effects against various diseases, including cancers, diabetes, nervous system disorders, and cardiovascular diseases [119,120,121]. Research has demonstrated that under hypoxic conditions, puerarin significantly and dose-dependently inhibits the production of ROS in PAECs [20] and PASMCs [21] in vitro. However, the mechanisms by which puerarin protects against ROS during pulmonary arterial remodeling remain unclear. In other diseases or non-vascular organs, puerarin seemed to suppress ROS production by targeting related enzymes or signaling molecules, such as NADPH, NOX4, or the nuclear factor E2-related factor 2 (Nrf2) pathway [122,123]. In contrast, its role in pulmonary arterial remodeling, whether it involves direct scavenging of ROS or inhibition of ROS generation, requires further investigation.

*Rutin*, a flavonol abundant in many plants, possesses notable antioxidant properties, effectively neutralizing oxidative species. It has been used to treat various diseases associated with ROS [124]. Rutin could suppress ROS generation in PAECs by modulating both mitochondrial and NOX4 pathways under hypoxic conditions. It also inhibits hypoxia-induced migration of PAECs and downregulates the expression of proliferating cell nuclear antigen, a marker of cell proliferation involved in the pathological remodeling of pulmonary arteries in PH [54]. A recent study indicated that rutin can interact with PKC alpha and suppress ferroptosis in PH [53]. Given that ROS is a key character of ferroptosis, the antioxidant effect of rutin may be partly attributable to its anti-ferroptosis activity. However, research on rutin in PH remains limited, and all English-language reports currently originate from the same research team. Therefore, the therapeutic potential of rutin in PH warrants further investigation.

*Proanthocyanidins* are oligomers or polymers of monomeric flavan-3-ols, representing the terminal products of the flavonoid biosynthetic pathway. They exhibit a variety of biological activities, including cardioprotective, antioxidant, neuroprotective, and anticancer properties [125]. Grape seed procyanidin extract, a complex of polyphenolic flavonoids rich in oligomeric proanthocyanidins, has been shown to reduce ROS production in PASMCs and restore the balance between superoxide dismutase (SOD) and malondialdehyde (MDA) in hypoxic rats. It also downregulated NOX4 expression in PASMCs and lung tissues from rats with HPH [62,125]. Regrettably, research on proanthocyanidins has stagnated in recent years.

*Chrysin*, a natural flavone found in many plants, as well as in honey and propolis [126], ameliorates pulmonary arterial remodeling in HPH rats. It reversed oxidative stress markers such as ROS and MDA, and downregulated the expression of NOX4 [57].

*Isoliquiritigenin* (ISL), a chalcone compound derived from *Glycyrrhizae Radix*, exhibits broad biological activities including anti-inflammatory, antioxidant, and cardioprotective effects [127]. It is regarded as a modulator of the Nrf2 signaling pathway, which plays a crucial role in antioxidant regulation [128]. In HPH rats, ISL inhibited the upregulation of NOX4 in lung tissues, significantly increased the SOD level and MDA content in serum and lung tissue. These effects may underlie its ability to attenuate pulmonary artery pressure, PA wall thickening, and right ventricular hypertrophy, thereby inhibiting pulmonary vascular remodeling induced by hypoxia [74].

*Hesperidin*, a dihydroflavonoid abundantly present in citrus fruits, has demonstrated antioxidant activity across various disease models. In hypertensive rats, it effectively alleviates oxidative stress and attenuates cardiovascular remodeling [62]. In myocardial ischemia models, hesperidin protects cells from hydrogen peroxide-induced damage [129], reduces plasma lipid peroxidation markers, and enhances antioxidant enzyme activity [130]. Given its established antioxidant and cardiovascular protective properties, hesperidin has also attracted attention in the field of PH. Studies have shown that it inhibits platelet-derived growth factor-BB (PDGF-BB)-induced proliferation of PASMCs [81]. Furthermore, in MCT-induced PH rats, hesperidin significantly ameliorates the disease by suppressing pulmonary arterial remodeling through modulation of the NF-κB pathway [131]. *Neohesperidin*, structurally analogous to hesperidin, also exhibits antioxidant and anti-inflammatory effects [132]. It inhibits angiotensin II-induced vascular oxidative stress, inflammation, fibrosis, and vascular remodeling in hypertension [89].

The approved vascular protective drug diosmin is hydrolyzed by intestinal flora to release *diosmetin*, its true active form. This bioactive flavonoid has demonstrated notable antioxidant activity and the ability to suppress vascular remodeling in hypertensive rats [91], suggesting considerable potential for treating PH. The anti-PH promise of diosmetin not only informs new drug discovery efforts but also supports the exploration of indication expansion for diosmin.

In summary, although many flavonoids possess antioxidant properties and can inhibit vascular remodeling in cardiovascular diseases, their therapeutic efficacy in PH remains uncertain, and the feasibility of therapeutic strategies targeting oxidative stress in PH therapy still requires further investigation. In our research, we have observed that flavonoids with therapeutic effects on PH, especially on pulmonary arterial remodeling, can reduce the production of ROS. However, ROS inhibition alone may not be sufficient. This suggests that antioxidant activity is one of several mechanisms through which flavonoids exert their effects. Multiple targets are involved in the action of flavonoids against pulmonary arterial remodeling, and their antioxidant effects are crucial in preventing or delaying disease progression. The antioxidant effects are essential in preventing or postponing pulmonary arterial remodeling. Combining agents that inhibit pulmonary artery pressure with antioxidant flavonoids may represent a promising strategy for PH.

### 3.2. Flavonoids Suppress Inflammatory Responses in Pulmonary Arterial Remodeling

Inflammation serves as a critical driver of vascular remodeling in cardiovascular diseases [133]. In PH, pulmonary arterial remodeling demonstrates a close relationship with inflammatory factors regarded as circulating biomarkers reflecting pathological alterations in Pas [134,135]. Inflammation exacerbates functional and structural damage to pulmonary arterial cells [136,137], promotes ECM degradation [138], and interacts with other physiological and pathological processes during pulmonary vascular remodeling [139], accompanying the progression of PH [140].

**Figure 5:**
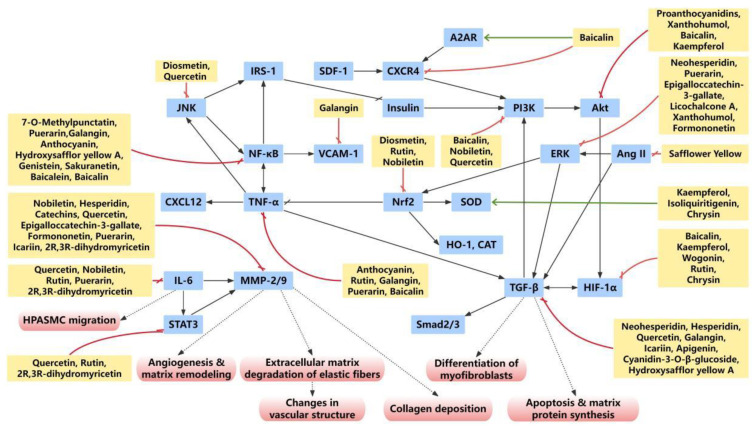
The inflammation-related molecular mechanism network of flavonoids in PH vascular remodeling. Various flavonoids regulate the process of vascular remodeling through multiple effects, multiple targets, and multiple pathways. Red box, biological effects; blue box, target; yellow box, flavonoids; red arrow, inhibition; green arrows, promotion.

Treatment with flavonoids in PH animal models consistently reduces levels of key inflammatory mediators, such as TNFα, interleukins, and transforming growth factor β (TGFβ). Therefore, the anti-inflammatory activity of flavonoids is considered one of the key mechanisms through which they inhibit pulmonary arterial remodeling (Figure 5).

#### 3.2.1. TNFα

TNFα is a pro-inflammatory cytokine implicated in vascular remodeling. In hypertensive rats, elevated levels of TNFα activate profibrotic mediators and promote the production of collagen and matrix proteins, thereby contributing to vascular structural changes [141,142]. In PH, TNFα further promotes pulmonary arterial remodeling by inducing PASMC proliferation [143] and EndoMT in PAECs [144]. It also causes overexpression of MMP-2/9, which mediates the degradation of elastic fibers in the ECM and facilitates alterations in vascular structure [145].

*Baicalin* and *baicalein*, two natural flavonoids discovered from the roots of *Scutellaria baicalensis Georgi*, are mutually convertible in vivo [43]. Both compounds have been widely reported to exert therapeutic effects in PH models, suppressing pulmonary arterial remodeling through multiple pathways. The anti-inflammatory activity has attracted particular attention. In MCT-induced PH rats, baicalin significantly ameliorated the condition by downregulating TNFα expression and upregulating BMPR2. In vitro, baicalin inhibited the proliferation of PASMCs induced by TNFα [37,146]. Also, baicalein has been shown to reduce TNFα levels during pulmonary arterial remodeling, exhibiting effects comparable to those of baicalin [147].

*Isorhamnetin*, a flavonoid extracted from *Hippophae rhamnoides* L., has demonstrated efficacy in an MCT-induced PH model by enhancing BMPR2 expression and suppressing inflammatory factors, including TNFα. In vitro, it also inhibited TNFα-induced PASMC proliferation [78].

*Galangin*, a natural flavonoid isolated from *Alpinia officinarum* Hance (*Zingiberaceae*), propolis, and honey, exhibits diverse pharmacological activities [148]. Its vascular protective effects have been extensively documented. This component inhibits VSMC proliferation by preventing the transition of the cell cycle from the G0/G1 phase to the S phase [148]. The adhesion factor VCAM-1 plays an important role in vascular inflammation and mediates vessel remodeling [92]. TNFα stimulates the NF-κB pathway, thereby activating VCAM-1 expression along with other pro-inflammatory molecules [149,150]. L-NAME is an L-arginine analogue and a non-selective NOS inhibitor. L-NAME chronically inhibits NO production, promotes sustained peripheral vasoconstriction, and leads to hypertension [151]. In L-NAME-induced hypertensive rats, galangin treatment significantly reduced TNF-R1 and VCAM-1 expression, suppressed NF-κB phosphorylation, lowered blood pressure, improved endothelium-dependent vasodilation, and inhibited aortic remodeling [92]. However, no report has yet investigated the effects of galangin on PH or pulmonary arterial remodeling.

*Fisetin*, a flavonoid commonly found in *Acacia greggii*, has been extensively studied for its effects on vascular cells, especially on aortic vessels through anti-inflammation mechanisms [152]. To date, there has been no report about fisetin on PH except for researchers who found that fisetin protected PAECs from lipopolysaccharide (LPS) injury by suppressing TNFα [153]. It is necessary to evaluate its potential role in modulating pulmonary arterial remodeling.

Several other flavonoids, such as ISL [74], *dihydromyricetin* [84], and *naringenin* [82], have also been reported to exert anti-inflammatory effects in PH animal models, notably by downregulating TNFα expression in lung tissues. Nevertheless, whether TNFα can serve as a novel therapeutic target for pulmonary arterial remodeling and PH remains a subject of ongoing research.

#### 3.2.2. TGFβ

Members of the TGFβ superfamily, including TGFβ, activin, and bone morphogenetic protein (BMP), are structurally related secretory cytokines conserved across a wide range of species [154]. Members of the TGFβ family play a crucial role in regulating diverse cellular processes, including proliferation, apoptosis, differentiation, and migration, and other functions. Upon binding to their specific receptors, they activate the Smad pathways. The resulting Smad complexes then regulate the transcription of target genes through synergistic interactions with various DNA-binding proteins and transcriptional co-activators or co-repressors [155,156,157]. TGFβ is particularly important in inflammatory response and promotes fibrosis, a key pathological feature of cardiovascular remodeling [158,159]. It also induces cell hypertrophy and stimulates ECM production [160,161] and has been implicated in Ang II-mediated VSMCs hypertrophy [162]. In PH, TGFβ1 is the one that has been the most extensively studied.

Under hypoxic conditions, TGFβ1 contributes to cellular adaptation and survival [163]. However, its sustained expression can impair bronchial epithelial repair while promoting the proliferation and migration of pulmonary arterial cells [164]. HIF-1α activates TGFβ1 signaling, and inhibition of HIF-1α has been shown to attenuate TGFβ1-induced proliferation and phenotypic switching in PASMCs [165,166]. Furthermore, TGFβ1 induces a hypertrophic and contractile smooth muscle phenotype via PI3K/Akt signaling [167] and promotes fibrotic responses through apoptosis stimulation and collagen synthesis [168,169,170]. Given its central role in these pathogenic mechanisms, therapeutic agents targeting the TGFβ1 signaling pathway generally demonstrate efficacy in mitigating vascular remodeling, particularly in the pulmonary arteries, and in treating PH.

*Icariin*, the primary active flavonoid of *Herba epimedii*, has demonstrated therapeutic effects on PH. In MCT-induced PH rats, icariin treatment alleviated hypertrophy and fibrosis in small Pas [47]. It is also able to inhibit phosphorylation and expression levels of downstream effectors of TGFβ1 signaling, including Smad2, Smad3, fibronectin, collagen, and proteoglycans, resulting in a rebalance of ECM and collagen deposition in lung tissue [171,172]. These mechanisms may underlie the significant inhibitory effect of icariin on pulmonary arterial remodeling in rats with PH.

*Apigenin*, a flavone abundantly present in various herbs and vegetables, including chamomile, thyme, and cruciferous plants, exhibits anti-proliferative and anti-inflammatory activities through multi-target mechanisms [173,174]. In bleomycin-induced pulmonary fibrosis models, oral administration of apigenin suppressed the upregulation of TGFβ [175]. However, studies on its therapeutic effects in PH remain limited. One study reported that apigenin promotes apoptosis of PASMC under hypoxic conditions via the HIF-1α/Kv1.5 channel pathway [75]. These findings imply that apigenin may also hold potential for treating other forms of PH, warranting further investigation.

#### 3.2.3. IL-6

The MCT-induced PH rat model is a classic inflammation-related experimental PH animal model that reflects the pathological process of “inflammation-vascular remodeling” [176]. In addition to TNFα and TGFβ, the abnormal expression of IL-6 is also observed in both circulating blood and lung tissues. The IL-6/interleukin-6 receptor (IL-6R) complex activates multiple signaling molecules, including STAT3, which upregulates MMP-9 expression in smooth muscle cells and contributes to pulmonary arterial remodeling [122]. Further supporting the pathogenic role of IL-6, transgenic mice overexpressing IL-6 spontaneously develop PH [177], underscoring its importance in disease progression. Notably, several flavonoids with demonstrated anti-PH efficacy have been shown to reduce the expression and release of IL-6 in PH animal models.

*Dihydromyricetin*, a major bioactive constituent of *Ampelopsis grossedentata*, exhibits anti-inflammatory, antioxidant, and anticancer effects [178,179]. Studies have demonstrated that dihydromyricetin suppresses STAT3 activation and MMP-9 expression in MCT-induced PH rats and also inhibits IL-6-induced migration of human pulmonary artery smooth muscle cells [84].

Although IL-6 is regarded as a key mediator of pulmonary arterial remodeling, only a limited number of flavonoids have been explicitly reported to inhibit remodeling via this pathway. Nonetheless, many flavonoids, including *hesperidin* [131], *baicalin* [37], and ISL [74], have been shown to reduce IL-6 levels in lung tissues. Whether IL-6 acts as a driver or a consequence of pulmonary vascular remodeling remains to be clarified through further experimental studies.

## 4. Flavonoids Target Specific Signaling Pathways Involved in Pulmonary Arterial Remodeling

### 4.1. BMPR2

BMPs are members of the TGFβ superfamily and play essential roles in early embryonic development, as well as in adult physiological processes. In mature organisms, BMP signaling helps maintain homeostasis in virtually all organ systems, including the cardiovascular system [180]. Two main types of transmembrane receptors mediate BMP signaling, BMPR1 and BMPR2 [181]. Within the pulmonary vasculature, BMPR2 is particularly critical in regulating vascular homeostasis. It was the first gene identified in association with heritable PH, with mutations detected in more than 70% of patients with hereditary PH and up to 20% of those diagnosed with idiopathic pulmonary arterial hypertension [182,183].

Functionally, BMPR2 acts as a negative regulator of smooth muscle cell proliferation and promotes endothelial cell survival [184], thereby helping to inhibit pathological vascular remodeling and preserve pulmonary arterial function in PH [185]. Conversely, loss-of-function mutations in BMPR2 impair BMP signaling, leading to abnormal vascular cell proliferation and accelerated fibrotic changes in the pulmonary vasculature [186]. BMPR2 deficiency is also linked to enhanced inflammatory responses, oxidative stress, and other PH-related pathological processes [187]. These findings collectively highlight the therapeutic potential of strategies aimed at restoring BMPR2 signaling for PH management.

*Quercetin*, a naturally occurring flavonoid, is commonly consumed in foods such as fruits, vegetables, nuts, and derived products like wine and chocolate. It has been shown to modulate multiple pathological processes involved in PH, including promoting vasodilation, inhibiting cell proliferation, and inducing apoptosis in PASMCs [188,189]. In MCT-induced PH rats, BMPR2 expression is significantly downregulated, accompanied by increased phosphorylation of Akt, as well as inhibition of Kv channel activity [190,191,192]. Quercetin treatment effectively restored BMPR2 expression, suppressed Akt phosphorylation, and partially recovered Kv currents. Through these mechanisms, quercetin reduced pulmonary arterial muscularization and attenuated vascular remodeling in PH models [193,194].

*Puerarin* protects human pulmonary artery endothelial cells (HPAECs) from hypoxia-induced injury by preventing the downregulation of BMPR2 [20]. However, its poor oral bioavailability has limited its clinical use to injectable formulations. To overcome this limitation, our lab has developed a novel crystal form of puerarin, Puer-V, which effectively addresses both its poor water solubility and inadequate lipid solubility. Puer-V demonstrates significantly improved gastrointestinal absorption and bioavailability, along with enhanced protective effects against PH [21]. In PH animal models, the BMPR2 signaling is substantially impaired, and Puer-V restores BMPR2 expression and reactivates its downstream pathway. Furthermore, Puer-V inhibits the abnormal proliferation of pulmonary arterial cells, improves vascular function, and attenuates pulmonary arterial remodeling and dysfunction. These findings position Puer-V as a promising novel therapeutic strategy for PH [22].

*Baicalin* exerts significant anti-inflammatory effects by downregulating TNFα expression. Since inflammation is a known trigger of BMPR2 dysfunction, and TNFα has been shown to promote PH by downregulating BMPR2 [37,146], baicalin’s anti-inflammatory action may help preserve BMPR2 signaling. In another study investigating the synergistic modulation of BMPR2 and NF-κB pathways, baicalin was found to inhibit NF-κB and Gremlin-1 (a BMP antagonist), while enhancing the expression of BMP2/4/9 and phospho-Smad1/5/8. These findings suggest that baicalin upregulates the BMPR2 signaling pathway, thereby attenuating pulmonary arterial remodeling [38].

### 4.2. PPARγ

PPARs are ligand-activated nuclear transcription factors implicated in key mechanisms of PH, including oxidative stress, inflammation, and vascular regulation. Among the three PPAR subtypes (PPARα, β/δ, and γ), PPARγ is expressed in endothelial cells and demonstrates therapeutic potential for PH. Its activation reduces ET-1 levels [195] and enhances NO production via the PI3K/Akt pathway [196,197].

*Puerarin* effectively protects HPAECs from hypoxia-induced injury by activating both BMPR2 and PPARγ signaling pathways while suppressing oxidative stress. Research indicates that PPARγ expression is significantly downregulated in the lung tissues of PH patients [198]. Under hypoxic conditions, PPARγ levels decline in endothelial cells; however, pre-treatment with puerarin can restore its expression. Puerarin may further activate the PI3K/Akt pathway through PPARγ, leading to enhanced eNOS activity and increasing NO production, collectively attenuating pulmonary arterial remodeling [20].

*Procyanidin*, a polyphenolic flavonoid commonly extracted from grape seeds and skins, has been shown to inhibit PASMC proliferation and pulmonary arterial remodeling in a cigarette smoke-induced PH rat model. This effect is mediated via the PPARγ/COX-2 pathway, with grape seed procyanidin extract significantly upregulating PPARγ expression in lung tissues of PH rats [63].

### 4.3. MAPK/ERK

MAPK signaling is critically involved in the growth response of PASMCs [199,200]. Increased MAPK activity is associated with pulmonary arterial remodeling [200]. ERK, a serine/threonine kinase belonging to the MAPK family, regulates key cellular processes including proliferation and apoptosis [201]. In patients with chronic obstructive pulmonary disease (COPD), structural alterations in the pulmonary arteries are characterized by vascular wall thickening and intimal remodeling of the small pulmonary arteries [202]. Additionally, significantly elevated levels of p-ERK are observed in the walls of small pulmonary arteries in COPD patients, suggesting that this protein may play a role in vascular remodeling [203]. Similarly, in both hypoxia-induced PH rat models and PDGF-BB-stimulated PASMCs, p-ERK levels are markedly increased [204]. Collectively, these findings demonstrate that activation of the MAPK/ERK pathway plays a critical role in the pathogenesis of pulmonary hypertension, highlighting its potential as a therapeutic target for future drug development.

*Coptidis Rhizoma*, derived from the dried rhizome of Coptis species, is a traditional Chinese herb with recognized potential for treating PH. Its principal flavonoids, including berberine and quercetin, effectively inhibit the proliferation and migration of PASMCs by downregulating the expression of MAPK1, NOX4, and cytochrome P450 1B1 [189]. In traditional Chinese medicine, Coptis has a long history of use and is frequently incorporated into herbal formulations for various ailments. One such formula, Sanhuang Jiaxin Decoction, composed of *Rhubarb*, *Coptis*, and *Scutellaria baicalensis*, has been shown to ameliorate PH by inhibiting PDE5 overexpression and promoting the expression of soluble guanylate cyclase subunits α1 and β1. This mechanism helps regulate cGMP levels and attenuate pulmonary vascular remodeling [205,206], which is likely attributable to the synergistic actions of its constituent flavonoids.

The ethyl acetate fraction of the hot water extract of *Salicornia europaea* L. is rich in flavonoids, such as p-coumaric acid, quercetin-3-β-D-glucoside, and isorhamnetin-3-β-D-glucoside [207]. It significantly and dose-dependently inhibits PDGF-BB-induced VSMC migration and proliferation, as well as the phosphorylation of MAPK, including p38 MAPK and ERK1/2 [207].

Upon activation by ROS, p38 MAPK not only promotes vascular cell proliferation and impairs PAEC function but also further enhances ROS production via NADPH oxidase [208]. This interaction forms a self-sustaining pathogenic cycle that exacerbates pulmonary vascular remodeling and endothelial dysfunction. Studies have shown that methanol extracts from *Mimosa pigra* leaves effectively suppress p38 MAPK activation in hypoxic PH models, thereby disrupting this detrimental feedback loop, providing endothelial protection, and significantly attenuating pulmonary vascular remodeling [209].

*Formononetin* (FMn), a natural phytoestrogen derived from *Trifolium pratense*, exhibits multiple biological activities, including proapoptotic, anti-inflammatory, and antitumor properties [210]. In the lungs of MCT-induced PH rats, elevated levels of p-ERK are observed, and inhibition of p-ERK has been shown to prevent pulmonary vascular remodeling [72]. FMn significantly attenuates MCT-induced ERK activation and partially ameliorates PH [210]. Notably, the ERK signaling pathway participates in regulating inflammatory response and ECM deposition [72], both of which are critical to the development of pulmonary vascular remodeling [210]. ECM deposition results from an interaction between ECM component synthesis and proteolysis, a process closely associated with MMPs [211]. Furthermore, FMn suppresses the MCT-induced upregulation of MMP-2/9, thereby reducing ECM accumulation and delaying PH progression, and alleviating right ventricular hypertrophy and pulmonary vascular remodeling [72]. FMn exhibits multi-target therapeutic effects in PH, positioning it as a promising candidate for drug development.

*Xanthohumol*, a polyphenolic compound found in the female inflorescences of hops and classified as an isoprenylated flavonoid, exhibits direct anti-angiogenic and pro-apoptotic effects on both endothelial and smooth muscle cells [212]. Intake of xanthohumol-enriched beer products has been shown to inhibit abnormal pulmonary vessel proliferation, correlating with the downregulation of ERK1/2 protein levels [213]. Additionally, xanthohumol promotes apoptosis by disrupting the PI3K/Akt pathway and modulating the expression of the anti-apoptotic protein B-cell lymphoma-extra large (BCL-XL). This action helps restore the balance between cell survival and apoptosis in pulmonary vascular tissues affected by PH, thereby reducing pathological vascular remodeling [213]. Xanthohumol-enriched beer represents a novel concept that could guide the development of flavonoid-based health foods for managing PH.

### 4.4. Nrf2

Nrf2 is a key transcription factor that orchestrates the cellular defense response against oxidative stress. The cellular defense mechanism is initiated by increasing the activity of the transcription factor Nrf2 [214]. Upon exposure to reactive ROS, the Nrf2 signaling pathway is activated, leading to the nuclear translocation of Nrf2. Inside the nucleus, Nrf2 promotes the transcription of antioxidant enzymes, such as HO-1, SOD, and catalase (CAT), which collectively mitigate oxidative damage [215,216]. Activation of the Nrf2 pathway has been shown to attenuate oxidative stress, organ damage, and inflammatory responses in MCT-induced PH rats [217].

HO-1, one of the key downstream effectors of Nrf2, catalyzes the degradation of heme into biliverdin, carbon monoxide, and free iron, thereby exerting antioxidant and anti-inflammatory effects [218]. *Diosmetin* has been reported to protect against LPS-induced acute lung injury in mice by activating the Nrf2/HO-1 axis and suppressing inflammatory responses [219]. In L-NAME-induced hypertensive rats, the expression of Nrf2 and HO-1 proteins is significantly downregulated, whereas diosmin treatment effectively restores their expression. This restoration is associated with the normalization of systemic oxidative stress markers (e.g., O^2−^ and MDA) and antioxidant enzymes (SOD and CAT) [91].

*Nobiletin*, a polymethoxyflavone found exclusively in citrus peel, exhibits a wide range of biological activities [220,221]. In L-NAME-induced hypertensive rats, nobiletin ameliorates vascular morphological abnormalities, reduces VSMC proliferation and collagen deposition, and attenuates oxidative stress. Furthermore, it restores the impaired expression of eNOS, Nrf2, and HO-1 proteins [222]. These results suggest that nobiletin exerts antihypertensive and vascular-protective effects, likely through the reactivation of the Nrf2/HO-1 signaling pathway.

### 4.5. NF-κB

NF-κB, a member of the transcription factor family, is significantly activated in the endothelial cells of patients with idiopathic PH, as well as in SU5416-induced and chronic hypoxia PH rat models [223]. It contributes extensively to multiple pathological processes in PH by exacerbating vascular inflammation and remodeling. Under hypoxic conditions or in response to inflammatory cytokines (e.g., TNFα and IL-6) and damage-associated molecular patterns, the IκB kinase complex promotes IκB degradation, leading to NF-κB nuclear translocation. This process enhances the release of pro-inflammatory mediators while reducing regulatory T cells, thereby aggravating perivascular inflammation [223,224]. Furthermore, hypoxia-activated mammalian target of rapamycin complex 1 induces NF-κB phosphorylation, thereby upregulating the expression of NF-κB target genes in PASMCs and promoting vascular remodeling [225]. NF-κB can also induce endothelial-to-mesenchymal transition, characterized by decreased CD31 and VE-cadherin expression and increasing α-SMA and collagen deposition, which leads to intimal thickening.

Experimental studies in rats show that L-NAME significantly upregulates the expression of p-NF-κB, TNF-R1, and VCAM-1. *Galangin* treatment effectively counteracts these changes [226]. TNFα, a pro-inflammatory cytokine, promotes vascular alterations by activating NF-κB and upregulating molecules such as VCAM-1 [227]. In the vascular system, TNFα stimulates NF-κB, which in turn activates VCAM-1 and other pro-inflammatory molecules [149,150,228]. Galangin treatment group exhibited significantly reduced plasma TNFα levels and ameliorated aortic remodeling, including wall thickening and fibrosis, in hypertensive rats [92].

*Diosmetin*, a citrus flavonoid with recognized antioxidant and anti-inflammatory properties, exerts anti-inflammatory effects by reducing IL-6 accumulation and suppressing the overexpression of p-JNK and p-NF-κB proteins in the aorta. In L-NAME-induced hypertensive rats, diosmetin administration effectively inhibits the upregulation of p-JNK and p-NF-κB.

Hypertensive diabetic rats exhibit significant endothelial dysfunction and cardiac/vascular hypertrophy, associated with impaired NO bioavailability and upregulation of Ang II/ROS/NF-κB inflammatory pathway [229]. In vitro, *puerarin* mitigates Ang II-induced endothelial dysfunction [230] and suppresses NF-κB activation and TNFα expression in the aortas of hypertensive diabetic rats [231].

### 4.6. PI3K/Akt

The PI3K/Akt signaling pathway serves as a critical driver of pulmonary vascular remodeling. It promotes proliferation, migration, and inflammatory responses in both PASMCs and PAECs, ultimately contributing to right ventricular hypertrophy and vascular remodeling [232]. This pathway further enhances remodeling by facilitating ubiquitin-mediated degradation of cAMP-response element binding protein and through synergistic interactions with multiple inflammatory signaling cascades [233].

*Baicalin* exhibits diverse pharmacological properties. It effectively lowers PAP, alleviates hypoxia-induced right ventricular hypertrophy and pulmonary congestion, and attenuates remodeling of PAs. Baicalin significantly reduces the arterial wall thickness-to-total thickness ratio compared to hypoxic model groups [42]. These protective effects are partially mediated through enhancing A2AR activity and downregulation of the PI3K/Akt signaling pathway.

*Nobiletin*’s potent anti-angiogenic activity in human endothelial cells was first identified in 2011, mediated through induction of cell cycle arrest and modulation of the VEGF pathway [234]. In 2023, the therapeutic potential of nobiletin was further demonstrated in pulmonary hypertension animal models. Studies showed that nobiletin reduces mPAP and attenuates pulmonary arterial remodeling in MCT-induced PH rats by regulating the PI3K/Akt/STAT3 pathway, while also decreasing levels of inflammatory cytokines, including IL-6, IL-1β, and TNF-α [85]. However, current research on nobiletin’s effects in PH remains limited, warranting further investigation.

*Kaempferol*, a natural flavonoid found in various Chinese herbs and vegetables, exhibits anti-proliferative and pro-apoptotic properties. In HPH rats, kaempferol decreased PAP and vascular remodeling. Mechanistically, it reduced phosphorylation of Akt and glycogen synthase kinase-3β, leading to downregulation of pro-proliferative and anti-apoptotic proteins, thereby inhibiting PASMC proliferation and ameliorating HPH [69].

The balance between cell viability and apoptosis is critical for tissue homeostasis [235], and the PI3K/Akt pathway plays a central role in maintaining this equilibrium [236]. Phosphorylated Akt inactivates components of the intrinsic apoptosis cascade (e.g., Bax, caspase-3, and caspase-9) and upregulates anti-apoptotic BCL-2 family proteins (e.g., BCL-2 and BCL-XL), conferring an apoptosis-resistant phenotype [237]. Long-term intake of *xanthohumol*-enriched beer has been shown to modulate PH by inhibiting Akt activation and downstream apoptotic factors, as well as suppressing BCL-XL expression, thereby reducing abnormal proliferation and apoptosis resistance in pulmonary vascular smooth muscle cells [213].

## 5. Flavonoids Regulate the Function of PA

**Figure 6:**
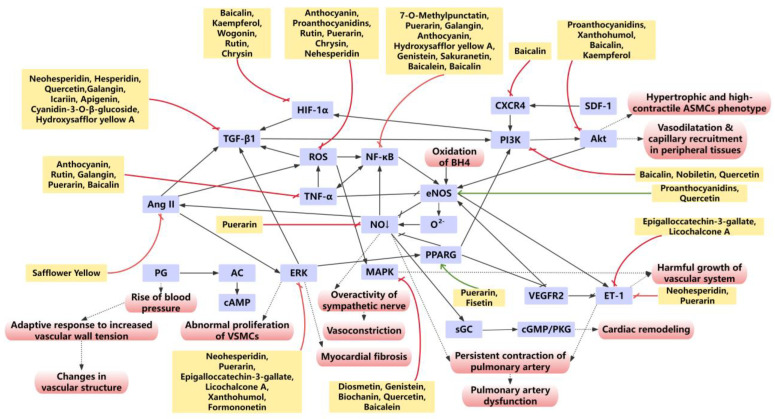
The main molecular mechanism network of PA function regulation of flavonoids in PH vascular remodeling. Various flavonoids regulate the process of vascular remodeling through multiple effects, multiple targets, and multiple pathways. Red box, biological effects; purple box, target; yellow box, flavonoids; red arrow, inhibition; green arrows, promotion.

Under physiological conditions, vascular activities, such as contraction and relaxation, are regulated by multiple coordinated factors. Endothelial injury disrupts the homeostatic balance of key endothelial-derived mediators, including ET, NO, and prostaglandin, which normally work in concert to maintain normal vascular physiological function. The imbalance is characterized by diminished anticoagulant properties, upregulated expression of adhesion molecules (e.g., E-selectin, ICAM-1, VCAM-1), and increased release of chemokines, cytokines, and growth factors, collectively leading to impaired vascular function [238]. Additionally, endothelium-dependent vasodilation plays a crucial role in regulating PAs and maintaining PAP. By modulating these endothelial factors, flavonoids may exert therapeutic effects against PH (Figure 6).

### 5.1. NO and ET-1

NO serves as a critical vasodilator in the pulmonary vasculature. PAECs synthesize NO via eNOS, thereby regulating vascular tone [239]. In PH patients, NO production is significantly diminished, resulting in impaired vasodilation, which, in turn, promotes vasoconstriction and pulmonary arterial remodeling [240]. Conversely, ET, a potent endogenous vasoconstrictor, shows elevated plasma levels in PH patients, establishing it as a pivotal driver of disease pathogenesis [241].

ET-1 and NO exhibit an antagonistic effect in vascular regulation. The combined increase in ET-1 levels and reduction in NO synergistically promote pulmonary arterial remodeling in PH. Diminished NO induces a phenotypic shift in PASMCs from a contractile to a synthetic state, thereby enhancing their proliferation and migration while inhibiting apoptosis [239]. Furthermore, reduced NO bioavailability facilitates fibroblast activation, leading to increased secretion of TGFβ and MMPs, which promotes collagen deposition and vascular wall fibrosis. ET-1 further activates fibroblasts and upregulates inflammatory mediators such as NF-κB and IL-6, sustaining a chronic inflammatory state that exacerbates vascular remodeling [242].

*Puerarin* ameliorates endothelial injury by enhancing the response of eNOS-induced NO to different stimuli [243], rebalancing the hypoxia-induced disruption of NO and ET-1, and thereby reducing the abnormal contraction of the PA. Pre-incubation with puerarin significantly increased NO level and decreased ET-1 level in HPAECs under hypoxic conditions [20]. Similarly, a methanol extract from *Mimosa pigra* leaves, rich in *quercetin glycosides* with antioxidant and anti-inflammatory properties, induces endothelium-dependent NO-mediated relaxation in both rat aorta and pulmonary arteries, effectively modulating endothelial function [209]. Red wine contains various active compounds, such as quercetin, resveratrol, and catechins, that support endothelial function by stimulating endothelium-dependent relaxation through NO and NOS pathways [244]. *Baicalein* ameliorates MCT-induced PH in rats. Additionally, baicalein suppressed glycogen synthase kinase-3β/β-catenin/ET-1/ETAR pathway and improved endothelial dysfunction. It upregulates eNOS expression while downregulating ET-1 and ETAR expression [44].

### 5.2. PGI2

Prostaglandin I2 (PGI2), synthesized by vascular endothelial cells, serves as a critical regulator in pulmonary arterial remodeling [226]. A deficiency in PGI2 disrupts multiple antiproliferative pathways, thereby promoting medial hypertrophy. Moreover, impaired PGI2 signaling leads to excessive activation of Akt, which stimulates fibroblast activity and contributes to adventitial fibrosis [245].

*Puerarin* has been shown to accelerate the reendothelialization of injured arteries, suppress neointima formation, and increase PGI2 production, thereby exerting vasodilatory effects in a carotid arterial injury model [246]. However, to date, no studies have reported the role of puerarin in PH via the PGI2 pathway.

*Baicalin* has been demonstrated to alleviate endothelial cell injury in pregnancy-induced hypertension while elevating levels of PGI2. Studies further indicate that baicalin mitigates hypoxia-induced endothelial damage and Ang II-induced endothelial dysfunction [247,248]. Additionally, baicalin increases PGI2 levels in the myocardium of post-infarction rats, suggesting a potential cardioprotective mechanism mediated through PGI2 upregulation [249].

### 5.3. MMP-2/9

Vascular structural changes are regulated by a multitude of factors. Alterations in vascular structure induced by elevated blood pressure can be regarded as an adaptive response to increased wall tension, a process that is closely associated with ECM degradation. Enzymes such as MMP-2 and MMP-9 play critical roles in vascular remodeling by facilitating ECM remodeling. Furthermore, the degradation also activates inflammatory mediators that promote the proliferation and migration of PASMCs and fibroblasts, further accelerating the pathological process [242].

Experimental evidence shows that MMP-2 expression is significantly elevated in the lungs of MCT-induced PH rats. This effect can be reversed by *icariin* treatment [250]. Considering that icariin also inhibits the TGFβ1/Smad2/3 signaling pathway, it is plausible that its anti-remodeling effects are mediated by interfering with the crosstalk between this signaling axis and MMP-2.

### 5.4. Ca^2+^

Elevated intracellular calcium concentration ([Ca^2+^]_i_) is a key driver of PASMC proliferation, a process in which store-operated calcium entry (SOCE) plays a significant role.

*Chrysin* is a natural flavonoid found in fruits, vegetables, and medicinal plants, known for its cardioprotective, anti-inflammatory, and antioxidant properties, along with a favorable safety profile [251]. It has been demonstrated to inhibit SOCE through multiple mechanisms, thereby reducing PASMC proliferation and mitigating distal pulmonary arterial remodeling in PH [58].

## 6. Clinical Trials of Flavonoids in Cardiovascular Diseases

Flavonoids, as a class of widely occurring bioactive compounds, have demonstrated beneficial effects on cardiovascular health, supported by numerous clinical trials. Clinical investigations retrieved from the Cochrane Library reveal that research on flavonoid interventions dates back to at least 1994, including studies on micronized flavonoid fractions for chronic venous insufficiency [252]. From 2018 onward, clinical trials conducted in countries including Russia and France further confirmed that micronized purified flavonoid fraction effectively relieves lower limb discomfort, leg pain, and heaviness in chronic venous disease, leading to rapid and sustained improvement in quality of life [253]. In addition, dietary intake of flavonoid-rich foods also confers cardiovascular benefits. For example, apple peel [254] and dark cocoa [255,256] have been reported to improve endothelial function, vascular elasticity, and platelet function. Collectively, these clinical and observational studies underscore the positive impact of flavonoids on cardiovascular health.

## 7. The Dilemma and Solution of Developing Flavonoids into Anti-PH Drugs

Flavonoids typically exhibit poor water solubility and are easily oxidized, leading to a reduction in their biological activities [257]. Additionally, many flavonoids have low oral bioavailability, often necessitating higher administered doses. Moreover, the metabolism and excretion of flavonoids in vivo are highly complex, making it challenging to strike a balance between dosage selection, control of adverse effects, and therapeutic efficacy. These factors collectively contribute to the challenges in the development of these compounds.

To overcome the challenges associated with flavonoid development, researchers have adopted various approaches, such as chemical modification and the design of novel delivery systems, to improve the water solubility, stability, and targeted delivery of these compounds. When used in combination with other compounds, flavonoids can enhance the pharmacological efficacy at lower doses, thereby reducing toxicity and improving the therapeutic efficacy. Furthermore, biotransformation techniques can convert flavonoids into forms that are more easily absorbed and utilized. Additionally, the preparation of solid flavonoid compounds with different crystalline forms can optimize oral pharmacokinetic properties and influence pharmacological activity.

The structural modification of natural compounds is a key strategy for discovering new chemical entities or enhancing the druggability of natural products. Given that PDE5A inhibitors are promising candidates for treating PH, researchers synthesized derivatives of the PDE-inhibitory isoflavones osajin and pomiferin. These derivatives exhibited significant PDE5A inhibition and vasorelaxant effects in vitro [258]. Although in vivo data are still limited, this discovery motivates the further modification of flavonoids with the potential in PH therapy, aiming to develop novel compounds with superior efficacy and improved drug-likeness.

Advances in delivery systems have further supported the therapeutic use of flavonoids in PH. For instance, a co-delivery system for p53 and baicalein has been developed, which effectively targeted PASMCs and significantly alleviated experimental PH in animal models [147]. While numerous studies have reported the preparation of flavonoid nanoparticles to improve solubility or develop new inhalation formulations [259,260], such applications remain largely unexplored in PH. In our laboratory, we developed various baicalein-loaded carriers to create a nano-formulation for inhalation. The resulting baicalein nanoparticles exhibited markedly increased solubility and demonstrated efficacy in cellular assays at lower concentrations. However, the outcomes in subsequent animal models did not meet our expectations.

In the pharmaceutical industry, the study of polymorphism of active pharmaceutical ingredients has become a critical aspect of drug development. Polymorphism studies of solid drugs for PH have also attracted considerable attention. For instance, ambrisentan, an endothelin receptor antagonist used to treat PH, has been prepared in a novel polymorph (form II), which is considered a promising candidate for a more bioavailable solid dosage form [261]. Inspired by this, we optimized the Puerarin to obtain a novel crystal type V Puerarin named Puer-V. In PH animal models, Puer-V demonstrated more potent effects than crude puerarin [21].

Substantial research efforts have been devoted to the study of flavonoids. We believe that natural flavonoids hold significant potential for development into health products or adjuvant therapies, largely due to their specific inhibitory effects on pulmonary arterial remodeling. This warrants greater attention from researchers, who could seek inspiration from flavonoids during the drug R&D process for PH.

## 8. Discussion and Further Consideration

The recent redefinition of PH diagnostic criteria has expanded the recognized patient population, creating both opportunities and challenges for disease management and new drug development [4,262]. Future therapeutic strategies should also consider developing new treatments for patients with mPAP of 21–24 mmHg. This situation calls for more precise vascular modulation, placing higher and stricter demands on therapeutic approaches. Notably, pharmacological interventions targeting the mechanisms of PA may hold greater clinical promise than those focusing solely on vasorelaxation.

Pulmonary arterial remodeling is an important pathological feature of PH, a complex condition driven by structural or functional abnormalities that increase pulmonary vascular resistance. While current therapies primarily target single pathways to alleviate vasoconstriction of PAs, growing evidence indicates that effective treatment for this disease must address both vasoconstriction and pulmonary arterial remodeling. Pulmonary arterial remodeling involves dysfunction and phenotypic switch of various pulmonary arterial cells along with inflammation and oxidative stress. As these pathological processes are driven by multiple interconnected pathways, drugs with multi-targeted pharmacological profiles have emerged as promising therapeutic agents.

Flavonoids, as important active components of traditional Chinese medicine, have garnered significant attention in drug research and development. They exhibit a broad spectrum of pharmacological effects, particularly anti-inflammatory and antioxidant properties. They also modulate multiple signaling pathways, such as PI3K/Akt, NF-κB, Nrf2, MMPs, and TGFβ, which are commonly implicated in pulmonary arterial remodeling. Given this multi-target activity, flavonoids show great potential in countering pulmonary arterial remodeling in PH. Therefore, screening promising flavonoids is crucial for advancing the treatment of PH, and potentially even reversing the disease. Given the complex pathophysiology and detailed classification of pulmonary hypertension, along with the distinct mechanisms of action exhibited by various flavonoids, the selection of the most promising flavonoid for practical application should be tailored to the specific clinical context.

However, many flavonoids have poor intestinal absorption and are rapidly eliminated after oral administration, resulting in low bioavailability that severely limits their application and development. This may explain the reason why just a few flavonoids have been used in clinics. Therefore, during screening for promising flavonoids, greater attention should be paid to the relationship between their chemical structure and pharmacokinetic and pharmacodynamic properties. We could optimize the structure of promising flavonoids to improve either biological activity or bioavailability. Other promising strategies include improving drug delivery systems, modifying crystal type, designing prodrugs, or developing combination therapies and twin drugs. We believe that developing new anti-PH drugs based on flavonoids will ultimately benefit patients with PH.

## Figures and Tables

**Table 1:** Classification of flavonoid compounds.

Basic Parent Structure	
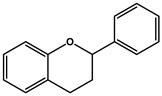	
Classification	
Flavone (R=H) Flavonol (R=OH)	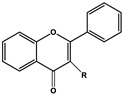
Dihydroflavone (R=H) Dihydroflavonol (R=OH)	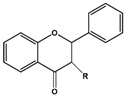
Isoflavone (R=H) Isoflavonol (R=OH)	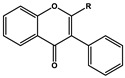
Dihydroisoflavone (R=H) Dihydroisoflavonol (R=OH)	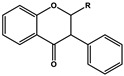
Chalcones	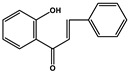
Aurones	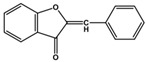
Flavanols	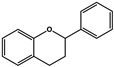
Anthocyanidins	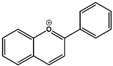

**Table 2:** Flavonoids studied in pulmonary arterial remodeling and are effective on PH models. The compounds are arranged in descending order according to the literature reports concerning pulmonary arterial remodeling and PH.

Name	Classification	Structure	Model	Dose for PH Animals	Targets on Vascular Remodeling	Refs.
Puerarin	Isoflavones	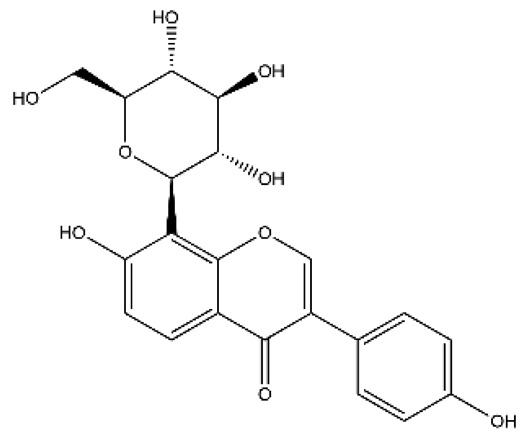	Monocrotaline (MCT) rats hypoxic pulmonary hypertension (HPH) mice/rats pulmonary arterial smooth muscle cell (PASMC), pulmonary artery endothelial cell (PAEC) cigarette smoke-exposed rats	p.o. 10–100 mg/kg/d i.p. 100 mg/kg/d	BMPR2/Smad, peroxisome proliferator-activated receptor (PPAR)-γ/phosphatidylinositol 3-kinase (PI3K)/protein kinase B (Akt), protein kinase C (PKC)	[20,21,22,23,24]
Breviscapine	Flavone	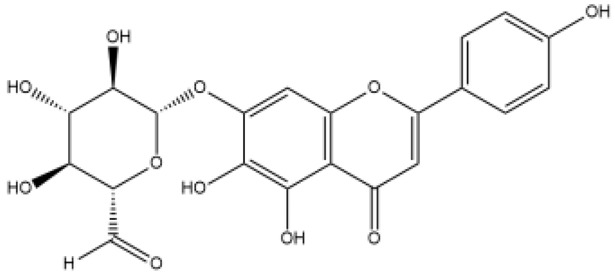	HPH rats PASMC	p.o. 60 mg/kg/d	PKC, fractalkine	[25,26]
Genistein	Isoflavones	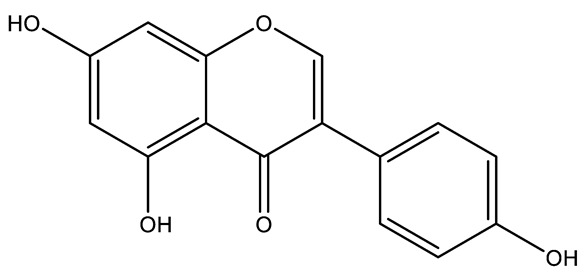	MCT rats HPH rats Low-temperature-induced PH in broiler chicks PAEC Isolated pulmonary artery	s.c. 20–200 μg, 1 mg/kg/d p.o. 20–60 mg/kg	PI3K/Akt/endothelial nitric oxide synthase (eNOS), estrogen receptor, β-adrenoceptor, erythropooietin/erythropoietin receptor, tyrosine kinases	[27,28,29,30,31,32,33,34]
Quercetin	Flavonol	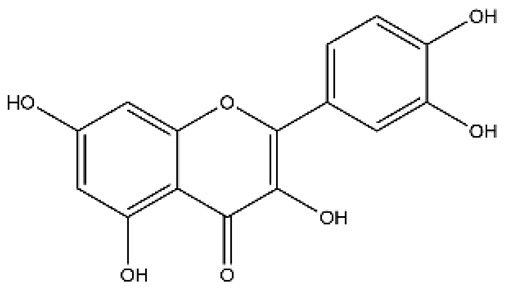	MCT rats HPH rats PASMC PAEC	i.p. 30 mg/kg/d p.o. 100 mg/kg/d	Poly ADP-ribose polymerase-1, miR-204, inositol-requiring enzyme 1α, Akt/extracellular signal-regulated kinase (ERK)1/2, forkhead box O1/mechanistic target of rapamycin, tropomyosin receptor kinase A	[26,34,35,36]
Baicalin	Flavone	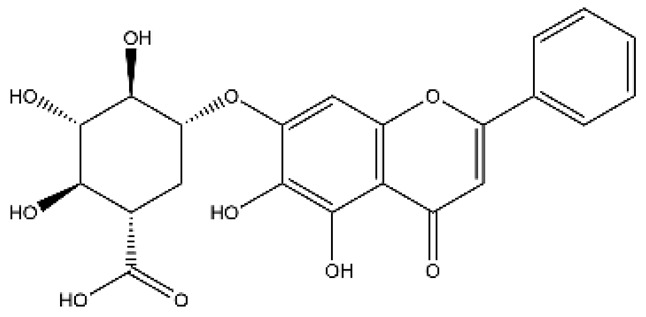	MCT rats HPH mice/rats PASMC PAEC	p.o. 25–100 mg/kg/d	calpain-1, PI3K/Akt/eNOS, peroxisome proliferator-activated receptor gamma coactivator 1α, BMPR2, tumor necrosis factor α (TNFα), ERK, nuclear factor kappa-B (NF-κB), Akt, hypoxia-inducible factor 1α (HIF1α)	[37,38,39,40,41,42]
Baicalein	Flavone	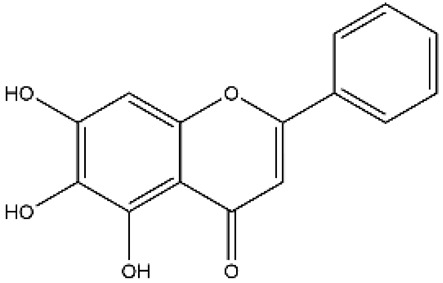	MCT rats Pneumonectomized rats PASMC	p.o. 50–100 mg/kg/d i.p. 10 mg/kg/d	NF-κB/BMPR2, endothelin (ET)-1/endothelin A receptor (ETAR), 12-lipoxygenase	[43,44,45,46]
Icariin	Flavonol	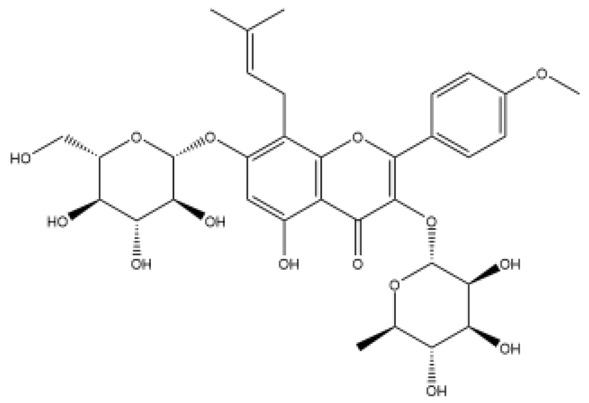	MCT rats	p.o. 20–100 mg/kg/d	TGFβ1, nitric oxide (NO)/cyclic guanosine monophosphate (cGMP)	[47,48]
Hydroxysafflor yellow A (Safflower Yellow)	Chalcones	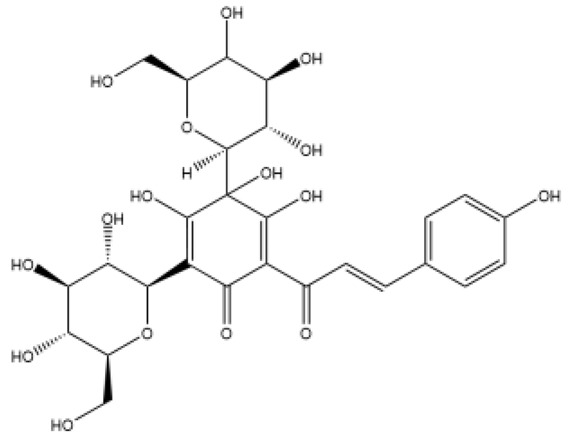	MCT rats HPH rats PASMC	i.p. 10 mg/kg/d p.o. 25–100 mg/kg/d	voltage-gated potassium (Kv) channel, inhibit inflammation and oxidative stress	[49,50,51,52]
Rutin	Flavonol	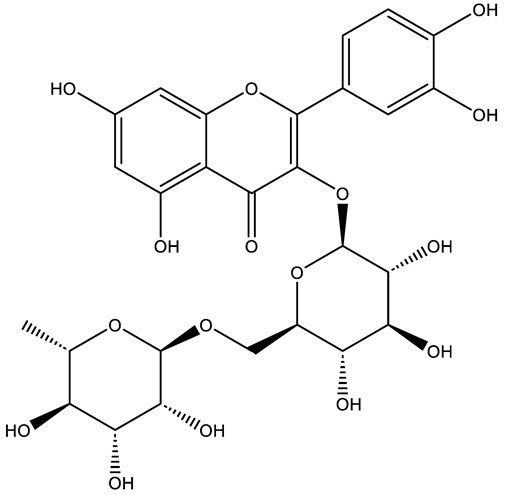	MCT rats PASMC PAEC	p.o. 200 mg/kg/d	PKCα, NADPH oxidase 4 (NOX4)	[53,54]
Chrysin	Flavone	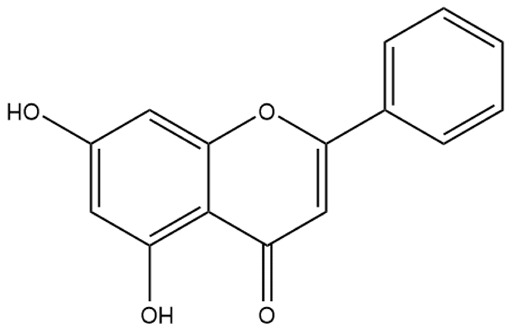	HPH rats MCT rats α-naphthylthiourea-PH rats PASMC	p.o. 10–100 mg/kg/d s.c. 50–100 mg/kg/d	Mitochondrial biogenesis, Ca^2+^ channel, vascular endothelial growth factor (VEGF), eNOS, NOX4	[55,56,57,58]
Epigallocatechin gallate (catechins, EGCG)	Flavanol	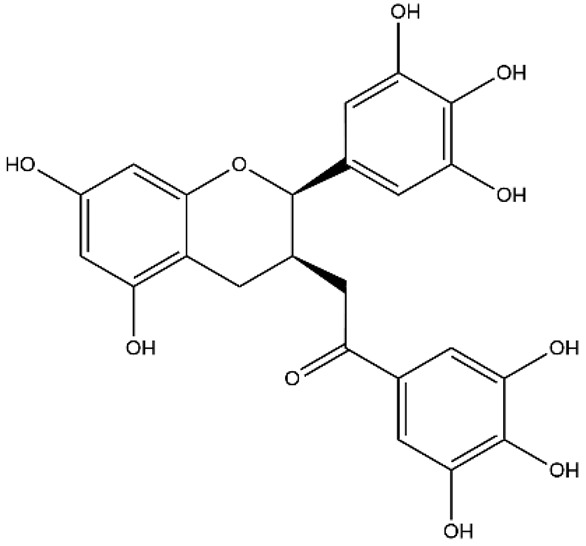	HPH rats PASMC Aortic smooth muscle cell	p.o. 50–200 mg/kg/d	Krüppel-like Factor 4/mitofusin 2/p-ERK	[59,60,61]
Proanthocyanidins	Anthocyanidins	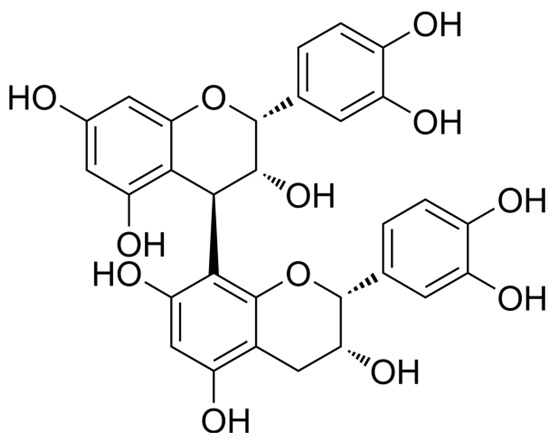	HPH rats MCT rats Cigarette-smoke-exposed rats PASMC	p.o. 250 mg/kg/d Endotracheal. 30 mg/kg	NOX4, PPARγ/cyclooxygenase-2 (COX-2), heat shock protein70, NF-κB	[62,63,64,65]
Luteolin	Flavone	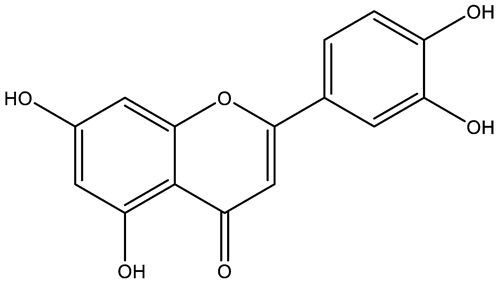	MCT rats HPH rats PASMC PAEC	p.o. 50–100 mg/kg/d	Arachidonic acid metabolites, HIF2α, PI3K/Akt/eNOS	[66,67,68]
Kaempferol	Flavonol	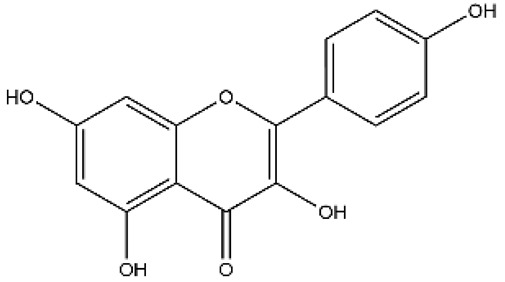	HPH rats MCT rats Isolated pulmonary artery	p.o. 25–150 mg/kg/d i.p. 150 mg/kg	Akt/glycogen synthase kinase 3β (GSK3β)/cyclin, Arachidonic acid metabolites; Direct vasorelaxation effects	[69,70,71]
Formononetin	Isoflavones	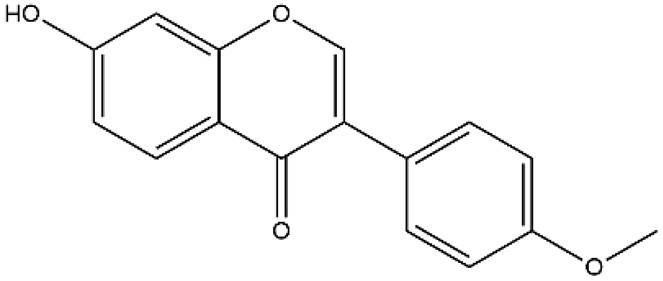	MCT rats Aortic smooth muscle cell	10–60 mg/kg/d	ERK, NF-κB, mitogen-activated protein kinase (MAPK)	[72,73]
Isoliquiritigenin	Chalcones	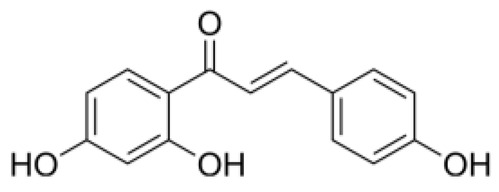	MCT rats	p.o. 10–20 mg/kg/d	Inhibit inflammation and proliferation	[74]
Apigenin	Flavone	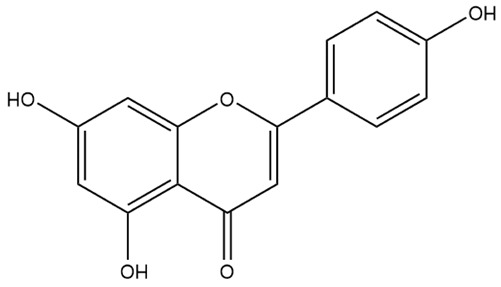	HPH rats PASMC	p.o. 50–100 mg/kg/d	HIF1α/Kv1.5	[75]
Pinocembrin	Dihydroflavone	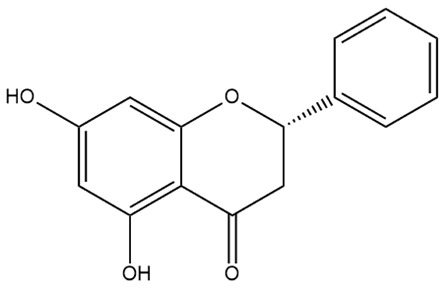	MCT rats	i.p. 50 mg/kg/d	Improves the therapeutic efficacy of endothelial progenitor cells in PH rats; inhibits atrial fibrillation	[76,77]
Isorhamnetin	Flavonol	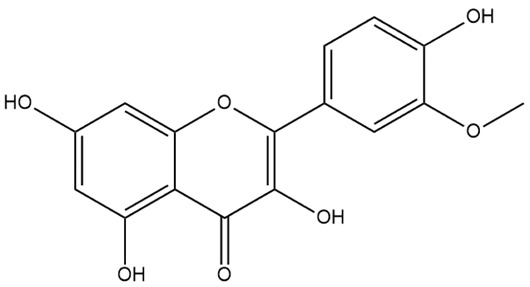	MCT rats PASMC	p.o. 50–150 mg/kg/d	BMPR2	[78]
Wogonin	Flavone	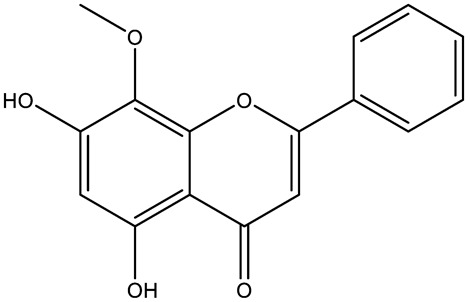	PASMC	-	HIF1/NOX4	[79,80]
Isoquercitrin	Flavonol	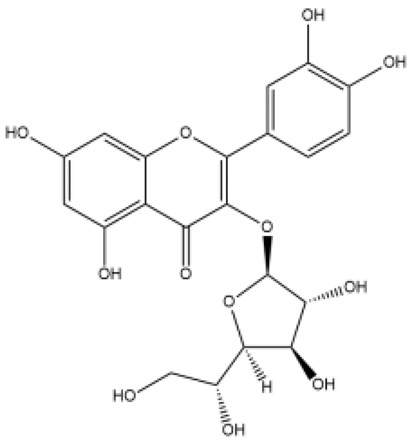	MCT rats PASMC	0.1% in feed for 3 weeks	Platelet-derived growth factor receptor β	[74]
Hesperidin	Dihydroflavone	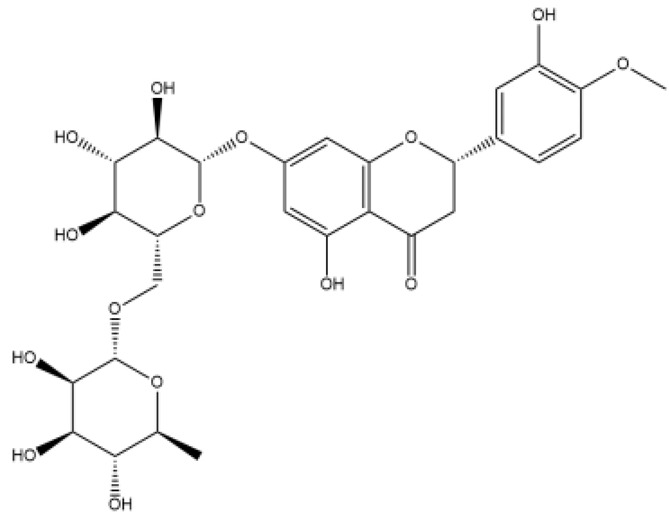	PASMC	-	Akt/GSK3β	[81]
Naringenin	Flavanones	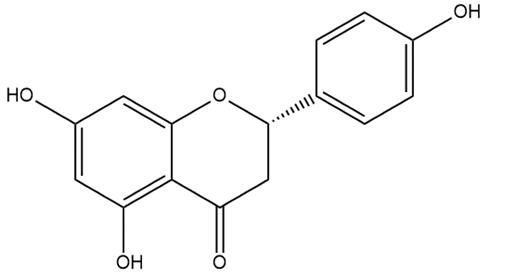	MCT rats	p.o. 50 mg/kg/d	Adds to the protective effect of L-arginine in PH rats	[82]
Naringin	Flavanones	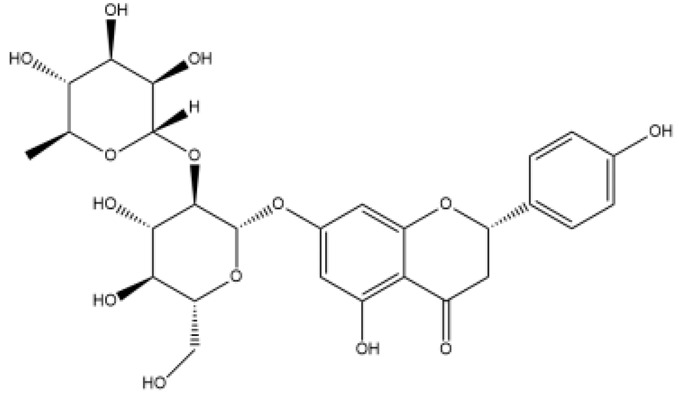	MCT rats PASMC	p.o. 25–100 mg/kg/d	ERK, NF-κB	[68]
Chalcone 4	Chalcones	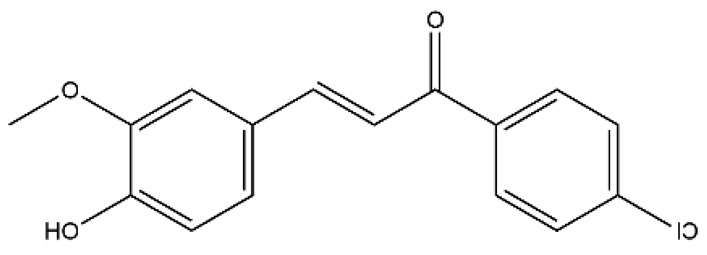	MCT rats HPH rats PASMC Pulmonary pericyte	i.p. 100 mg/kg/d	C-X-C motif chemokine ligand 12	[83]
Dihydromyricetin	Flavanonols	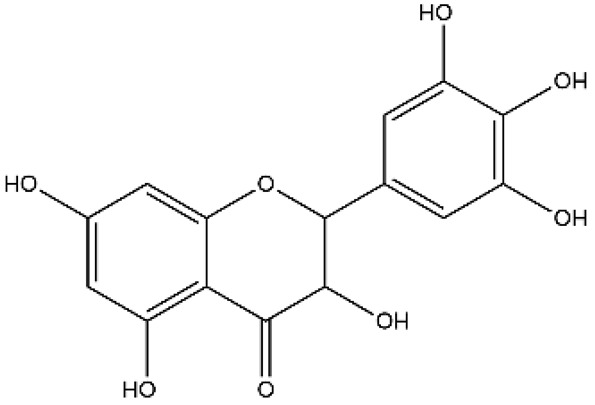	MCT rats PASMC	p.o. 100 mg/kg/d	Signal transducer and activator of transcription 3 (STAT3)/matrix metallopeptidase (MMP)-9, interleukin-6 (IL-6)	[84]
Nobiletin	Flavone	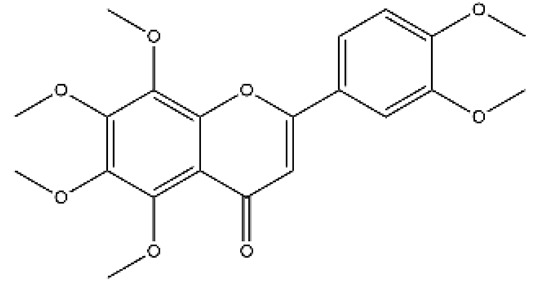	MCT rats	p.o. 1–10 mg/kg	PI3K/Akt/STAT3	[85]

Explanation of Abbreviations: oral administration (p.o.); intraperitoneal injection (i.p.); subcutaneous Injections (s.c.).

**Table 3:** Flavonoids that have inhibitory effects on vascular remodeling without literature reports about PH.

Name	Classification	Structure	Model	Dose for Animals	Targets on Vascular Remodeling	Refs.
Fisetin	Flavonol	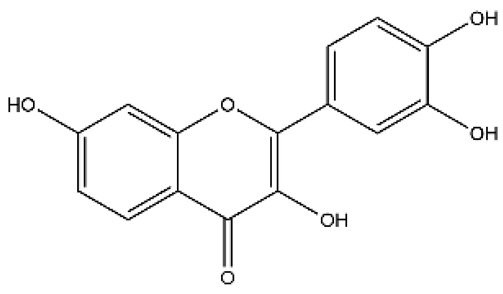	Aged rodent (old mice) Carotid balloon injury model in spontaneously hypertensive rats human aortic endothelial cells vascular smooth muscle cell (VSMC)	p.o. 100 mg/kg/d (Intermittent) i.p. 3 mg/kg/d	Improves arterial function by decreasing cellular senescence; attenuate neointimal formation through PPARγ/paraoxonase 2 pathway	[86,87,88]
Neohesperidin	Dihydroflavone	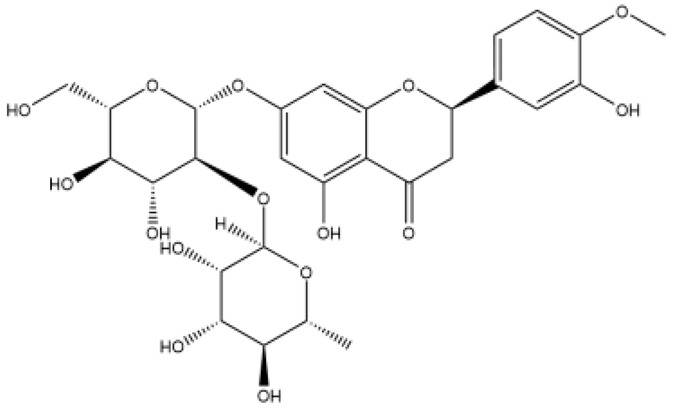	Angiotensin II (Ang II)-induced hypertension mice	i.v. 50 mg/kg	As antioxidant and could inhibit angiotensin II induced hypertension and vascular remodeling	[89]
Diosmetin	Flavone	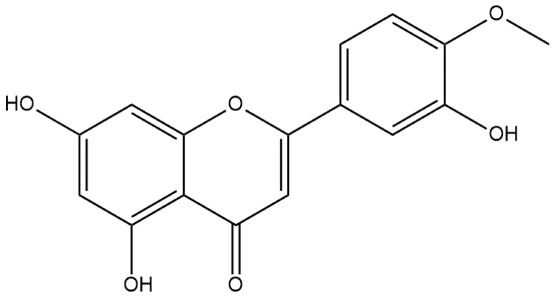	N-Nitro-L-arginine methyl ester (L-NAME) induced hypertension rats	p.o. 20, 40 mg/kg	Ca^2+^ channel antagonism, potassium channel activation and antimuscarinic receptor-linked vasodilatory effects; Nuclear factor erythroid 2–related factor (Nrf2)/heme oxygenase-1 (HO-1), c-Jun N-terminal kinase (JNK)/NF-κB	[90,91]
Galangin	Flavonol	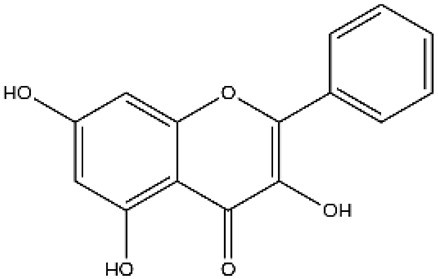	L-NAME induced hypertension rats Isolated mesenteric vessels	p.o. 30–60 mg/kg/d	Tumor necrosis factor receptor 1 (TNF-R1), p-NF-κB, vascular cell adhesion molecule-1 (VCAM-1)	[92]
7-O-Methylpunctatin	Isoflavone	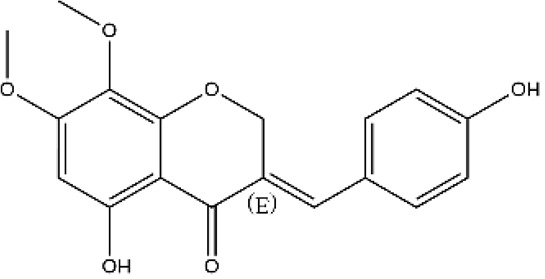	Arterial smooth muscle cell	-	NF-κB	[93]
Licochalcone A	Chalcones	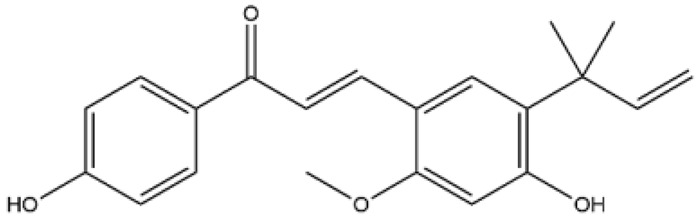	VSMC	-	ERK1/2	[94]

Explanation of Abbreviations: oral administration (p.o.); intraperitoneal injection (i.p.); intravenous Injection (i.v.).

## Data Availability

No data was used for the research described in the article.

## Abbreviations

Akt	protein kinase B
Ang II	angiotensin II
BCL-XL	B-cell lymphoma-extra large
BMP	bone morphogenetic protein
BMPR	bone morphogenetic protein receptor
CAT	catalase
CD31	endothelial cell adhesion molecule-1
cGMP	cyclic guanosine monophosphate
COPD	chronic obstructive pulmonary disease
COX-2	cyclooxygenase-2
ECM	extracellular matrix
EndoMT	endothelial-mesenchymal transition
eNOS	endothelial nitric oxide synthase
ERK	extracellular signal-regulated kinase
ET	endothelin
ETAR	endothelin A receptor
FMn	formononetin
GSK3β	glycogen synthase kinase 3β
HIF	hypoxia-inducible factor
HO-1	heme oxygenase-1
HPAEC	human pulmonary artery endothelial cell
HPH	hypoxic pulmonary hypertension
HPV	hypoxic pulmonary vasoconstriction
IL-1β	interleukin-1β
IL-6	interleukin-6
IL-6R	interleukin-6 receptor
ISL	isoliquiritigenin
JNK	C-Jun N-terminal Kinase
Kv	voltage-gated potassium
L-NAME	N-Nitro-L-arginine methyl ester
LPS	lipopolysaccharide
MAPK	mitogen-activated protein kinase
MCT	monocrotaline
MDA	malondialdehyde
MMP	matrix metalloprotein
mPAP	mean pulmonary arterial pressure
NADPH	nicotinamide adenine dinucleotide phosphate
NF-κB	nuclear factor kappa-B
NO	nitric oxide
NOX4	NADPH oxidase 4
Nrf2	nuclear factor E2-related factor 2
PA	pulmonary artery
PAEC	pulmonary artery endothelial cell
PASMC	pulmonary arterial smooth muscle cell
PDE5	phosphodiesterase type 5
PDGF-BB	platelet-derived growth factor-BB
PGI2	prostaglandin I2
PH	pulmonary hypertension
PI3K	phosphatidylinositol 3-kinase
PKC	protein kinase C
PPAR	peroxisome proliferator-activated receptor
ROS	reactive oxygen species
sGC	soluble guanylate cyclase
SOCE	store-operated calcium entry
SOD	superoxide dismutase
STAT3	transducer and activator of transcription 3
TGFβ	transforming growth factor β
TNF-R1	tumor necrosis factor receptor 1
TNFα	tumor necrosis factor α
VCAM-1	vascular cell adhesion molecule-1
VEGF	vascular endothelial growth factor
VSMC	vascular smooth muscle cell
WSPH	World Symposia on Pulmonary Hypertension
α-SMA	α-smooth muscle actin
[Ca^2+^]_i_	intracellular calcium concentration

## References

[1] Mocumbi A., Humbert M., Saxena A., Jing Z.C., Sliwa K., Thienemann F., Archer S.L., Stewart S. (2024). Pulmonary Hypertension. Nat. Rev. Dis. Primers.

[2] Moutchia J., McClelland R.L., Al-Naamani N., Appleby D.H., Holmes J.H., Minhas J., Mazurek J.A., Palevsky H.I., Ventetuolo C.E., Kawut S.M. (2024). Pulmonary Arterial Hypertension Treatment: An Individual Participant Data Network Meta-Analysis. Eur. Heart J..

[3] Condon D.F., Nickel N.P., Anderson R., Mirza S., de Jesus Perez V.A. (2019). The 6th World Symposium on Pulmonary Hypertension: What’s Old Is New. F1000Research.

[4] Humbert M., Kovacs G., Hoeper M.M., Badagliacca R., Berger R.M.F., Brida M., Carlsen J., Coats A.J.S., Escribano-Subias P., Ferrari P. (2022). 2022 Esc/Ers Guidelines for the Diagnosis and Treatment of Pulmonary Hypertension. Eur. Heart J..

[5] Auth R., Klinger J.R. (2023). Emerging Pharmacotherapies for the Treatment of Pulmonary Arterial Hypertension. Expert. Opin. Investig. Drugs.

[6] Humbert M., Guignabert C., Bonnet S., Dorfmüller P., Klinger J.R., Nicolls M.R., Olschewski A.J., Pullamsetti S.S., Schermuly R.T., Stenmark K.R. (2019). Pathology and Pathobiology of Pulmonary Hypertension: State of the Art and Research Perspectives. Eur. Respir. J..

[7] Zhang Z.Q., Zhu S.K., Wang M., Wang X.A., Tong X.H., Wan J.Q., Ding J.W. (2022). New Progress in Diagnosis and Treatment of Pulmonary Arterial Hypertension. J. Cardiothorac. Surg..

[8] Girerd B., Coulet F., Jaïs X., Eyries M., Van Der Bruggen C., De Man F., Houweling A., Dorfmüller P., Savale L., Sitbon O. (2015). Characteristics of Pulmonary Arterial Hypertension in Affected Carriers of a Mutation Located in the Cytoplasmic Tail of Bone Morphogenetic Protein Receptor Type 2. Chest.

[9] Ranasinghe A., Tennakoon T., Schwarz M.A. (2024). Emerging Epigenetic Targets and Their Molecular Impact on Vascular Remodeling in Pulmonary Hypertension. Cells.

[10] Wang Z., Zhang Y.X., Shi J.Z., Yan Y., Zhao L.L., Kou J.J., He Y.Y., Xie X.M., Zhang S.J., Pang X.B. (2024). Rna M6a Methylation and Regulatory Proteins in Pulmonary Arterial Hypertension. Hypertens. Res..

[11] Thompson A.A.R., Lawrie A. (2017). Targeting Vascular Remodeling to Treat Pulmonary Arterial Hypertension. Trends Mol. Med..

[12] Humbert M., McLaughlin V., Gibbs J.S.R., Gomberg-Maitland M., Hoeper M.M., Preston I.R., Souza R., Waxman A., Escribano Subias P., Feldman J. (2021). Sotatercept for the Treatment of Pulmonary Arterial Hypertension. N. Engl. J. Med..

[13] Joshi S.R., Atabay E.K., Liu J., Ding Y., Briscoe S.D., Alexander M.J., Andre P., Kumar R., Li G. (2023). Sotatercept Analog Improves Cardiopulmonary Remodeling and Pulmonary Hypertension in Experimental Left Heart Failure. Front. Cardiovasc. Med..

[14] Morse A., Cheng T.L., Peacock L., Mikulec K., Little D.G., Schindeler A. (2016). Rap-011 Augments Callus Formation in Closed Fractures in Rats. J. Orthop. Res..

[15] Shen N., Wang T., Gan Q., Liu S., Wang L., Jin B. (2022). Plant Flavonoids: Classification, Distribution, Biosynthesis, and Antioxidant Activity. Food Chem..

[16] Bailly C. (2024). Pharmacological Properties of Extracts and Prenylated Isoflavonoids from the Fruits of Osage Orange (Maclura Pomifera (Raf.) C.K.Schneid.). Fitoterapia.

[17] Singh A., Singh J., Parween G., Khator R., Monga V. (2025). A Comprehensive Review of Apigenin a Dietary Flavonoid: Biological Sources, Nutraceutical Prospects, Chemistry and Pharmacological Insights and Health Benefits. Crit. Rev. Food Sci. Nutr..

[18] Zhao Z., Yang X., Zhang Y. (1998). Clinical Study of Puerarin in Treatment of Patients with Unstable Angina. Zhongguo Zhong Xi Yi Jie He Za Zhi.

[19] Guo J., Wang Y., Li J., Zhang J., Wu Y., Wang G. (2023). Overview and Recent Progress on the Biosynthesis and Regulation of Flavonoids in *Ginkgo biloba* L. Int. J. Mol. Sci..

[20] Yuan T., Zhang H., Chen D., Chen Y., Lyu Y., Fang L., Du G. (2019). Puerarin Protects Pulmonary Arteries from Hypoxic Injury through the Bmprii and Pparγ Signaling Pathways in Endothelial Cells. Pharmacol. Rep..

[21] Chen D., Zhang H.F., Yuan T.Y., Sun S.C., Wang R.R., Wang S.B., Fang L.H., Lyu Y., Du G.H. (2022). Puerarin-V Prevents the Progression of Hypoxia- and Monocrotaline-Induced Pulmonary Hypertension in Rodent Models. Acta Pharmacol. Sin..

[22] Chen C., Chen C., Wang Z., Wang L., Yang L., Ding M., Ding C., Sun Y., Lin Q., Huang X. (2012). Puerarin Induces Mitochondria-Dependent Apoptosis in Hypoxic Human Pulmonary Arterial Smooth Muscle Cells. PLoS ONE.

[23] Zhang X., Liu Q., Zhang C., Sheng J., Li S., Li W., Yang X., Wang X., He S., Bai J. (2019). Puerarin Prevents Progression of Experimental Hypoxia-Induced Pulmonary Hypertension Via Inhibition of Autophagy. J. Pharmacol. Sci..

[24] Zhu Z., Xu Y., Zou H., Zhang Z., Ni W., Chen S. (2008). Effects of Puerarin on Pulmonary Vascular Remodeling and Protein Kinase C-Alpha in Chronic Cigarette Smoke Exposure Smoke-Exposed Rats. J. Huazhong Univ. Sci. Technol. Med. Sci..

[25] Chen X.J., Cheng D.Y., Yang L., Xia X.Q., Guan J. (2006). Effect of Breviscapine on Fractalkine Expression in Chronic Hypoxic Rats. Chin. Med. J..

[26] Gao J., Chen G., He H., Liu C., Xiong X., Li J., Wang J. (2017). Therapeutic Effects of Breviscapine in Cardiovascular Diseases: A Review. Front. Pharmacol..

[27] Chen Y., Chen D., Liu S., Yuan T., Guo J., Fang L., Du G. (2019). Systematic Elucidation of the Mechanism of Genistein against Pulmonary Hypertension Via Network Pharmacology Approach. Int. J. Mol. Sci..

[28] Zhang M., Wu Y., Wang M., Wang Y., Tausif R., Yang Y. (2018). Genistein Rescues Hypoxia-Induced Pulmonary Arterial Hypertension through Estrogen Receptor and Β-Adrenoceptor Signaling. J. Nutr. Biochem..

[29] Zheng Z., Yu S., Zhang W., Peng Y., Pu M., Kang T., Zeng J., Yu Y., Li G. (2017). Genistein Attenuates Monocrotaline-Induced Pulmonary Arterial Hypertension in Rats by Activating Pi3k/Akt/Enos Signaling. Histol. Histopathol..

[30] Kuriyama S., Morio Y., Toba M., Nagaoka T., Takahashi F., Iwakami S., Seyama K., Takahashi K. (2014). Genistein Attenuates Hypoxic Pulmonary Hypertension Via Enhanced Nitric Oxide Signaling and the Erythropoietin System. Am. J. Physiol. Lung Cell Mol. Physiol..

[31] Matori H., Umar S., Nadadur R.D., Sharma S., Partow-Navid R., Afkhami M., Amjedi M., Eghbali M. (2012). Genistein, a Soy Phytoestrogen, Reverses Severe Pulmonary Hypertension and Prevents Right Heart Failure in Rats. Hypertension.

[32] Henno P., Maurey C., Danel C., Bonnette P., Souilamas R., Stern M., Delclaux C., Lévy M., Israël-Biet D. (2009). Pulmonary Vascular Dysfunction in End-Stage Cystic Fibrosis: Role of Nf-Kappab and Endothelin-1. Eur. Respir. J..

[33] Homma N., Morio Y., Takahashi H., Yamamoto A., Suzuki T., Sato K., Muramatsu M., Fukuchi Y. (2006). Genistein, a Phytoestrogen, Attenuates Monocrotaline-Induced Pulmonary Hypertension. Respiration.

[34] Rajabi S., Najafipour H., Jafarinejad Farsangi S., Joukar S., Beik A., Iranpour M., Kordestani Z. (2020). Perillyle Alcohol and Quercetin Ameliorate Monocrotaline-Induced Pulmonary Artery Hypertension in Rats through Parp1-Mediated Mir-204 Down-Regulation and Its Downstream Pathway. BMC Complement. Med. Ther..

[35] Cao X., He Y., Li X., Xu Y., Liu X. (2019). The Ire1α-Xbp1 Pathway Function in Hypoxia-Induced Pulmonary Vascular Remodeling, Is Upregulated by Quercetin, Inhibits Apoptosis and Partially Reverses the Effect of Quercetin in Pasmcs. Am. J. Transl. Res..

[36] Huang S., Zhu X., Huang W., He Y., Pang L., Lan X., Shui X., Chen Y., Chen C., Lei W. (2017). Quercetin Inhibits Pulmonary Arterial Endothelial Cell Transdifferentiation Possibly by Akt and Erk1/2 Pathways. Biomed. Res. Int..

[37] Xue X., Zhang S., Jiang W., Wang J., Xin Q., Sun C., Li K., Qi T., Luan Y. (2021). Protective Effect of Baicalin against Pulmonary Arterial Hypertension Vascular Remodeling through Regulation of Tnf-A Signaling Pathway. Pharmacol. Res. Perspect..

[38] Zhang Z., Zhang L., Sun C., Kong F., Wang J., Xin Q., Jiang W., Li K., Chen O., Luan Y. (2017). Baicalin Attenuates Monocrotaline-Induced Pulmonary Hypertension through Bone Morphogenetic Protein Signaling Pathway. Oncotarget.

[39] Jiang H.X., Wang X.D., Wang H.X., Liu T. (2023). Baicalin Attenuates Pulmonary Vascular Remodeling by Inhibiting Calpain-1 Mediated Endothelial-to-Mesenchymal Transition. Heliyon.

[40] Fujiwara T., Takeda N., Hara H., Ishii S., Numata G., Tokiwa H., Katoh M., Maemura S., Suzuki T., Takiguchi H. (2023). Pgc-1α-Mediated Angiogenesis Prevents Pulmonary Hypertension in Mice. JCI Insight.

[41] Yan G., Wang J., Yi T., Cheng J., Guo H., He Y., Shui X., Wu Z., Huang S., Lei W. (2019). Baicalin Prevents Pulmonary Arterial Remodeling in Vivo Via the Akt/Erk/Nf-Κb Signaling Pathways. Pulm. Circ..

[42] Huang X., Wu P., Huang F., Xu M., Chen M., Huang K., Li G.P., Xu M., Yao D., Wang L. (2017). Baicalin Attenuates Chronic Hypoxia-Induced Pulmonary Hypertension Via Adenosine a(2a) Receptor-Induced Sdf-1/Cxcr4/Pi3k/Akt Signaling. J. Biomed. Sci..

[43] Cui L., Yuan T., Zeng Z., Liu D., Liu C., Guo J., Chen Y. (2022). Mechanistic and therapeutic perspectives of baicalin and baicalein on pulmonary hypertension: A comprehensive review. Biomed. Pharmacother..

[44] Hsu W.L., Lin Y.C., Jeng J.R., Chang H.Y., Chou T.C. (2018). Baicalein Ameliorates Pulmonary Arterial Hypertension Caused by Monocrotaline through Downregulation of Et-1 and Et(a)R in Pneumonectomized Rats. Am. J. Chin. Med..

[45] Shi R., Wei Z., Zhu D., Fu N., Wang C., Yin S., Liang Y., Xing J., Wang X., Wang Y. (2018). Baicalein Attenuates Monocrotaline-Induced Pulmonary Arterial Hypertension by Inhibiting Vascular Remodeling in Rats. Pulm. Pharmacol. Ther..

[46] Preston I.R., Hill N.S., Warburton R.R., Fanburg B.L. (2006). Role of 12-Lipoxygenase in Hypoxia-Induced Rat Pulmonary Artery Smooth Muscle Cell Proliferation. Am. J. Physiol. Lung Cell Mol. Physiol..

[47] Xiang Y., Cai C., Wu Y., Yang L., Ye S., Zhao H., Zeng C. (2020). Icariin Attenuates Monocrotaline-Induced Pulmonary Arterial Hypertension Via the Inhibition of Tgf-Β1/Smads Pathway in Rats. Evid. Based Complement. Altern. Med..

[48] Li L.S., Luo Y.M., Liu J., Zhang Y., Fu X.X., Yang D.L. (2016). Icariin Inhibits Pulmonary Hypertension Induced by Monocrotaline through Enhancement of NO/cGMP Signaling Pathway in Rats. Evid. Based Complement. Alternat Med..

[49] Ji X.Y., Lei C.J., Kong S., Li H.F., Pan S.Y., Chen Y.J., Zhao F.R., Zhu T.T. (2024). Hydroxy-Safflower Yellow a Mitigates Vascular Remodeling in Rat Pulmonary Arterial Hypertension. Drug Des. Devel Ther..

[50] Han X., Zhang Y., Zhou Z., Zhang X., Long Y. (2016). Hydroxysafflor Yellow a Improves Established Monocrotaline-Induced Pulmonary Arterial Hypertension in Rats. J. Int. Med. Res..

[51] Li L., Dong P., Hou C., Cao F., Sun S., He F., Song Y., Li S., Bai Y., Zhu D. (2016). Hydroxysafflor Yellow a (Hsya) Attenuates Hypoxic Pulmonary Arterial Remodelling and Reverses Right Ventricular Hypertrophy in Rats. J. Ethnopharmacol..

[52] Bai Y., Lu P., Han C., Yu C., Chen M., He F., Yi D., Wu L. (2012). Hydroxysafflor Yellow a (Hsya) from Flowers of *Carthamus tinctorius* L. And Its Vasodilatation Effects on Pulmonary Artery. Molecules.

[53] Che H., Yi J., Zhao X., Yu H., Wang X., Zhang R., Li X., Fu J., Li Q. (2024). Characterization of Pkcα-Rutin Interactions and Their Application as a Treatment Strategy for Pulmonary Arterial Hypertension by Inhibiting Ferroptosis. Food Funct..

[54] Li Q., Qiu Y., Mao M., Lv J., Zhang L., Li S., Li X., Zheng X. (2014). Antioxidant Mechanism of Rutin on Hypoxia-Induced Pulmonary Arterial Cell Proliferation. Molecules.

[55] Kobayashi T., Kim J.D., Naito A., Yanagisawa A., Jujo-Sanada T., Kasuya Y., Nakagawa Y., Sakao S., Tatsumi K., Suzuki T. (2022). Multi-Omics Analysis of Right Ventricles in Rat Models of Pulmonary Arterial Hypertension: Consideration of Mitochondrial Biogenesis by Chrysin. Int. J. Mol. Med..

[56] Dong F., Zhang J., Chen X., Zhang S., Zhu L., Peng Y., Guo Z. (2020). Chrysin Alleviates Monocrotaline-Induced Pulmonary Hypertension in Rats through Regulation of Intracellular Calcium Homeostasis in Pulmonary Arterial Smooth Muscle Cells. J. Cardiovasc. Pharmacol..

[57] Li X.W., Wang X.M., Li S., Yang J.R. (2015). Effects of Chrysin (5,7-Dihydroxyflavone) on Vascular Remodeling in Hypoxia-Induced Pulmonary Hypertension in Rats. Chin. Med..

[58] Dong F., Zhang J., Zhu S., Lan T., Yang J., Li L. (2019). Chrysin Alleviates Chronic Hypoxia-Induced Pulmonary Hypertension by Reducing Intracellular Calcium Concentration in Pulmonary Arterial Smooth Muscle Cells. J. Cardiovasc. Pharmacol..

[59] Zhu T.T., Zhang W.F., Luo P., He F., Ge X.Y., Zhang Z., Hu C.P. (2017). Epigallocatechin-3-Gallate Ameliorates Hypoxia-Induced Pulmonary Vascular Remodeling by Promoting Mitofusin-2-Mediated Mitochondrial Fusion. Eur. J. Pharmacol..

[60] Shu Z., Yu M., Zeng G., Zhang X., Wu L., Tan X. (2014). Epigallocatechin-3-Gallate Inhibits Proliferation of Human Aortic Smooth Muscle Cells Via up-Regulating Expression of Mitofusin 2. Eur. J. Cell Biol..

[61] Chowdhury A., Sarkar J., Chakraborti T., Chakraborti S. (2015). Role of Spm-Cer-S1p Signalling Pathway in Mmp-2 Mediated U46619-Induced Proliferation of Pulmonary Artery Smooth Muscle Cells: Protective Role of Epigallocatechin-3-Gallate. Cell Biochem. Funct..

[62] Jin H., Liu M., Zhang X., Pan J., Han J., Wang Y., Lei H., Ding Y., Yuan Y. (2016). Grape Seed Procyanidin Extract Attenuates Hypoxic Pulmonary Hypertension by Inhibiting Oxidative Stress and Pulmonary Arterial Smooth Muscle Cells Proliferation. J. Nutr. Biochem..

[63] Liu J., Hu S., Zhu B., Shao S., Yuan L. (2020). Grape Seed Procyanidin Suppresses Inflammation in Cigarette Smoke-Exposed Pulmonary Arterial Hypertension Rats by the Ppar-Γ/Cox-2 Pathway. Nutr. Metab. Cardiovasc. Dis..

[64] Chen F., Wang H., Zhao J., Yan J., Meng H., Zhan H., Chen L., Yuan L. (2019). Grape Seed Proanthocyanidin Inhibits Monocrotaline-Induced Pulmonary Arterial Hypertension Via Attenuating Inflammation: In Vivo and in Vitro Studies. J. Nutr. Biochem..

[65] Chen F., Wang H., Yan J., Lai J., Cai S., Yuan L., Zheng S. (2018). Grape Seed Proanthocyanidin Reverses Pulmonary Vascular Remodeling in Monocrotaline-Induced Pulmonary Arterial Hypertension by Down-Regulating Hsp70. Biomed. Pharmacother..

[66] Song K., Duan Q., Ren J., Yi J., Yu H., Che H., Yang C., Wang X., Li Q. (2022). Targeted Metabolomics Combined with Network Pharmacology to Reveal the Protective Role of Luteolin in Pulmonary Arterial Hypertension. Food Funct..

[67] Ji L., Su S., Xin M., Zhang Z., Nan X., Li Z., Lu D. (2022). Luteolin Ameliorates Hypoxia-Induced Pulmonary Hypertension Via Regulating Hif-2α-Arg-No Axis and Pi3k-Akt-Enos-No Signaling Pathway. Phytomedicine.

[68] Zuo W., Liu N., Zeng Y., Xiao Z., Wu K., Yang F., Li B., Song Q., Xiao Y., Liu Q. (2021). Luteolin Ameliorates Experimental Pulmonary Arterial Hypertension Via Suppressing Hippo-Yap/Pi3k/Akt Signaling Pathway. Front. Pharmacol..

[69] Zhang X., Yang Z., Su S., Nan X., Xie X., Li Z., Lu D. (2023). Kaempferol Ameliorates Pulmonary Vascular Remodeling in Chronic Hypoxia-Induced Pulmonary Hypertension Rats Via Regulating Akt-Gsk3β-Cyclin Axis. Toxicol. Appl. Pharmacol..

[70] Yi J., Wang X., Song K., Ren J., Che H., Yu H., Li Q. (2022). Integrated Metabolomics and Mechanism to Reveal the Protective Effect of Kaempferol on Pulmonary Arterial Hypertension. J. Pharm. Biomed. Anal..

[71] Mahobiya A., Singh T.U., Rungsung S., Kumar T., Chandrasekaran G., Parida S., Kumar D. (2018). Kaempferol-Induces Vasorelaxation Via Endothelium-Independent Pathways in Rat Isolated Pulmonary Artery. Pharmacol. Rep..

[72] Wu Y., Cai C., Yang L., Xiang Y., Zhao H., Zeng C. (2020). Inhibitory Effects of Formononetin on the Monocrotaline-Induced Pulmonary Arterial Hypertension in Rats. Mol. Med. Rep..

[73] Cai C., Xiang Y., Wu Y., Zhu N., Zhao H., Xu J., Lin W., Zeng C. (2019). Formononetin Attenuates Monocrotaline-Induced Pulmonary Arterial Hypertension Via Inhibiting Pulmonary Vascular Remodeling in Rats. Mol. Med. Rep..

[74] Jin H., Jiang Y., Du F., Guo L., Wang G., Kim S.C., Lee C.W., Shen L., Zhao R. (2019). Isoliquiritigenin Attenuates Monocrotaline-Induced Pulmonary Hypertension Via Inhibition of the Inflammatory Response and Pasmcs Proliferation. Evid. Based Complement. Alternat Med..

[75] He Y., Fang X., Shi J., Li X., Xie M., Liu X. (2020). Apigenin Attenuates Pulmonary Hypertension by Inducing Mitochondria-Dependent Apoptosis of Pasmcs Via Inhibiting the Hypoxia Inducible Factor 1α-Kv1.5 Channel Pathway. Chem. Biol. Interact..

[76] Yi Y., Tianxin Y., Zhangchi L., Cui Z., Weiguo W., Bo Y. (2023). Pinocembrin Attenuates Susceptibility to Atrial Fibrillation in Rats with Pulmonary Arterial Hypertension. Eur. J. Pharmacol..

[77] Ahmed L.A., Rizk S.M., El-Maraghy S.A. (2017). Pinocembrin Ex Vivo Preconditioning Improves the Therapeutic Efficacy of Endothelial Progenitor Cells in Monocrotaline-Induced Pulmonary Hypertension in Rats. Biochem. Pharmacol..

[78] Chang Z., Wang J.L., Jing Z.C., Ma P., Xu Q.B., Na J.R., Tian J., Ma X., Zhou W., Zhou R. (2020). Protective Effects of Isorhamnetin on Pulmonary Arterial Hypertension: In Vivo and in Vitro Studies. Phytother. Res..

[79] Li Y., Fu Y., Liu Y., Zhao D., Liu L., Bourouis S., Algarni A.D., Zhong C., Wu P. (2023). An Optimized Machine Learning Method for Predicting Wogonin Therapy for the Treatment of Pulmonary Hypertension. Comput. Biol. Med..

[80] Cui L., Zeng Z., Wang X., Yuan T., Wang C., Liu D., Guo J., Chen Y. (2023). Deciphering the Mechanism of Wogonin, a Natural Flavonoid, on the Proliferation of Pulmonary Arterial Smooth Muscle Cells by Integrating Network Pharmacology and in Vitro Validation. Curr. Issues Mol. Biol..

[81] Wei L., Deng W., Cheng Z., Guo H., Wang S., Zhang X., He Y., Tang Q. (2016). Effects of Hesperetin on Platelet-Derived Growth Factor-Bb-Induced Pulmonary Artery Smooth Muscle Cell Proliferation. Mol. Med. Rep..

[82] Ahmed L.A., Obaid A.A., Zaki H.F., Agha A.M. (2014). Naringenin Adds to the Protective Effect of L-Arginine in Monocrotaline-Induced Pulmonary Hypertension in Rats: Favorable Modulation of Oxidative Stress, Inflammation and Nitric Oxide. Eur. J. Pharm. Sci..

[83] Bordenave J., Thuillet R., Tu L., Phan C., Cumont A., Marsol C., Huertas A., Savale L., Hibert M., Galzi J.L. (2020). Neutralization of Cxcl12 Attenuates Established Pulmonary Hypertension in Rats. Cardiovasc. Res..

[84] Li Q., Wang J., Zhu X., Zeng Z., Wu X., Xu Y., Xie J., Yu J. (2017). Dihydromyricetin Prevents Monocrotaline-Induced Pulmonary Arterial Hypertension in Rats. Biomed. Pharmacother..

[85] Yin Q., Wang S., Yang J., Fan C., Yu Y., Li J., Mei F., Zhang S., Xi R., Zhang X. (2023). Nobiletin Attenuates Monocrotaline-Induced Pulmonary Arterial Hypertension through Pi3k/Akt/Stat3 Pathway. J. Pharm. Pharmacol..

[86] Mahoney S.A., Venkatasubramanian R., Darrah M.A., Ludwig K.R., VanDongen N.S., Greenberg N.T., Longtine A.G., Hutton D.A., Brunt V.E., Campisi J. (2024). Intermittent Supplementation with Fisetin Improves Arterial Function in Old Mice by Decreasing Cellular Senescence. Aging Cell.

[87] Pei F., Pei H., Su C., Du L., Wang J., Xie F., Yin Q., Gao Z. (2021). Fisetin Alleviates Neointimal Hyperplasia Via Pparγ/Pon2 Antioxidative Pathway in Shr Rat Artery Injury Model. Oxid. Med. Cell Longev..

[88] Kim S.G., Sung J.Y., Kang Y.J., Choi H.C. (2023). Pparγ Activation by Fisetin Mitigates Vascular Smooth Muscle Cell Senescence Via the Mtorc2-Foxo3a-Autophagy Signaling Pathway. Biochem. Pharmacol..

[89] Zhang J., Hui Y., Liu F., Yang Q., Lu Y., Chang Y., Liu Q., Ding Y. (2022). Neohesperidin Protects Angiotensin Ii-Induced Hypertension and Vascular Remodeling. Front. Pharmacol..

[90] Ahmad T., Javed A., Khan T., Althobaiti Y.S., Ullah A., Almutairi F.M., Shah A.J. (2022). Investigation into the Antihypertensive Effects of Diosmetin and Its Underlying Vascular Mechanisms Using Rat Model. Pharmaceuticals.

[91] Meephat S., Prasatthong P., Potue P., Bunbupha S., Pakdeechote P., Maneesai P. (2021). Diosmetin Ameliorates Vascular Dysfunction and Remodeling by Modulation of Nrf2/Ho-1 and P-Jnk/P-Nf-Κb Expression in Hypertensive Rats. Antioxidants.

[92] Chaihongsa N., Maneesai P., Sangartit W., Potue P., Bunbupha S., Pakdeechote P. (2021). Galangin Alleviates Vascular Dysfunction and Remodelling through Modulation of the Tnf-R1, P-Nf-Κb and Vcam-1 Pathways in Hypertensive Rats. Life Sci..

[93] Fardoun M., Iratni R., Dehaini H., Eid A., Ghaddar T., El-Elimat T., Alali F., Badran A., Eid A.H., Baydoun E. (2019). 7-O-Methylpunctatin, a Novel Homoisoflavonoid, Inhibits Phenotypic Switch of Human Arteriolar Smooth Muscle Cells. Biomolecules.

[94] Park J.H., Lim H.J., Lee K.S., Lee S., Kwak H.J., Cha J.H., Park H.Y. (2008). Anti-Proliferative Effect of Licochalcone a on Vascular Smooth Muscle Cells. Biol. Pharm. Bull..

[95] Hopkins S.R., Stickland M.K. (2023). The Pulmonary Vasculature. Semin. Respir. Crit. Care Med..

[96] Kolluru G.K., Glawe J.D., Pardue S., Kasabali A., Alam S., Rajendran S., Cannon A.L., Abdullah C.S., Traylor J.G., Shackelford R.E. (2022). Methamphetamine Causes Cardiovascular Dysfunction Via Cystathionine Gamma Lyase and Hydrogen Sulfide Depletion. Redox Biol..

[97] Gimbrone M.A., Topper J.N., Nagel T., Anderson K.R., Garcia-Cardeña G. (2000). Endothelial Dysfunction, Hemodynamic Forces, and Atherogenesis. Ann. N. Y Acad. Sci..

[98] Rubanyi G.M., Romero J.C., Vanhoutte P.M. (1986). Flow-Induced Release of Endothelium-Derived Relaxing Factor. Am. J. Physiol..

[99] Kuchan M.J., Frangos J.A. (1994). Role of Calcium and Calmodulin in Flow-Induced Nitric Oxide Production in Endothelial Cells. Am. J. Physiol..

[100] Benza R.L., Grünig E., Sandner P., Stasch J.P., Simonneau G. (2024). The Nitric Oxide-Soluble Guanylate Cyclase-Cgmp Pathway in Pulmonary Hypertension: From Pde5 to Soluble Guanylate Cyclase. Eur. Respir. Rev..

[101] Takahashi M., Murase M. (2024). Outcomes of High-Dose Inhaled Nitric Oxide and Oxygen Administration for Severe Pulmonary Hypertension with Bronchopulmonary Dysplasia. Cureus.

[102] Liu R., Yuan T., Wang R., Gong D., Wang S., Du G., Fang L. (2023). Insights into Endothelin Receptors in Pulmonary Hypertension. Int. J. Mol. Sci..

[103] Nikitopoulou I., Orfanos S.E., Kotanidou A., Maltabe V., Manitsopoulos N., Karras P., Kouklis P., Armaganidis A., Maniatis N.A. (2016). Vascular Endothelial-Cadherin Downregulation as a Feature of Endothelial Transdifferentiation in Monocrotaline-Induced Pulmonary Hypertension. Am. J. Physiol. Lung Cell Mol. Physiol..

[104] Gairhe S., Awad K.S., Dougherty E.J., Ferreyra G.A., Wang S., Yu Z.X., Takeda K., Demirkale C.Y., Torabi-Parizi P., Austin E.D. (2021). Type I Interferon Activation and Endothelial Dysfunction in Caveolin-1 Insufficiency-Associated Pulmonary Arterial Hypertension. Proc. Natl. Acad. Sci. USA.

[105] Feron O., Dessy C., Moniotte S., Desager J.P., Balligand J.L. (1999). Hypercholesterolemia Decreases Nitric Oxide Production by Promoting the Interaction of Caveolin and Endothelial Nitric Oxide Synthase. J. Clin. Investig..

[106] Marudamuthu A.S., Bhandary Y.P., Fan L., Radhakrishnan V., MacKenzie B., Maier E., Shetty S.K., Nagaraja M.R., Gopu V., Tiwari N. (2019). Caveolin-1-Derived Peptide Limits Development of Pulmonary Fibrosis. Sci. Transl. Med..

[107] Xiao L., Tong X. (2019). Advances in Molecular Mechanism of Vascular Remodeling in Pulmonary Arterial Hypertension. Zhejiang Da Xue Xue Bao Yi Xue Ban.

[108] Ranchoux B., Antigny F., Rucker-Martin C., Hautefort A., Péchoux C., Bogaard H.J., Dorfmüller P., Remy S., Lecerf F., Planté S. (2015). Endothelial-to-Mesenchymal Transition in Pulmonary Hypertension. Circulation.

[109] Klouda T., Kim Y., Baek S.H., Bhaumik M., Li Y., Liu Y., Wu J.C., Raby B.A., Perez V.J., Yuan K. (2025). Specialized Pericyte Subtypes in the Pulmonary Capillaries. EMBO J..

[110] Régent A., Ly K.H., Lofek S., Clary G., Tamby M., Tamas N., Federici C., Broussard C., Chafey P., Liaudet-Coopman E. (2016). Proteomic Analysis of Vascular Smooth Muscle Cells in Physiological Condition and in Pulmonary Arterial Hypertension: Toward Contractile Versus Synthetic Phenotypes. Proteomics.

[111] Badran A., Nasser S.A., Mesmar J., El-Yazbi A.F., Bitto A., Fardoun M.M., Baydoun E., Eid A.H. (2020). Reactive Oxygen Species: Modulators of Phenotypic Switch of Vascular Smooth Muscle Cells. Int. J. Mol. Sci..

[112] Zhang L., Chen Y., Li G., Chen M., Huang W., Liu Y., Li Y. (2016). Tgf-Β1/Fgf-2 Signaling Mediates the 15-Hete-Induced Differentiation of Adventitial Fibroblasts into Myofibroblasts. Lipids Health Dis..

[113] Niinimaki E., Muola P., Parkkila S., Kholová I., Haapasalo H., Pastorekova S., Pastorek J., Paavonen T., Mennander A. (2016). Carbonic Anhydrase Ix Deposits Are Associated with Increased Ascending Aortic Dilatation. Scand. Cardiovasc. J..

[114] (2021). Group of Pulmonary Embolism and Pulmonary Vascular Disease; Respiratory Disease Branch of Chinese Medical As-sociation; Working Committee of Pulmonary Embolism and Pulmonary Vascular Disease, Respiratory Physician Branch of Chinese Medical Association; National Collaborative Group on Prevention and Treatment of Pulmonary Embolism and Pulmonary Vascular Disease; National Expert Panel for the Standardized System Construction Project for Pulmonary Hypertension. Guidelines for Diagnosis and Treatment of Pulmonary Hypertension in China, 2021 Edition. Chin. Med. J..

[115] Pokharel M.D., Marciano D.P., Fu P., Franco M.C., Unwalla H., Tieu K., Fineman J.R., Wang T., Black S.M. (2023). Metabolic Reprogramming, Oxidative Stress, and Pulmonary Hypertension. Redox Biol..

[116] Rawat M., Lakshminrusimha S., Vento M. (2022). Pulmonary Hypertension and Oxidative Stress: Where Is the Link?. Semin. Fetal Neonatal Med..

[117] Wang J., Huang J., Wang L., Chen C., Yang D., Jin M., Bai C., Song Y. (2017). Urban Particulate Matter Triggers Lung Inflammation Via the Ros-Mapk-Nf-Κb Signaling Pathway. J. Thorac. Dis..

[118] Demarco V.G., Whaley-Connell A.T., Sowers J.R., Habibi J., Dellsperger K.C. (2010). Contribution of Oxidative Stress to Pulmonary Arterial Hypertension. World J. Cardiol..

[119] Cheng Y., Leng W., Zhang J. (2016). Protective Effect of Puerarin against Oxidative Stress Injury of Neural Cells and Related Mechanisms. Med. Sci. Monit..

[120] Chen T., Chen H., Wang Y., Zhang J. (2016). In Vitro and in Vivo Antitumour Activities of Puerarin 6″-O-Xyloside on Human Lung Carcinoma A549 Cell Line Via the Induction of the Mitochondria-Mediated Apoptosis Pathway. Pharm. Biol..

[121] Zhang Y., Wang H., Yu L., Chen J. (2015). The Puerarin Improves Renal Function in Stz-Induced Diabetic Rats by Attenuating Enos Expression. Ren. Fail..

[122] Zhang Q., Yao M., Qi J., Song R., Wang L., Li J., Zhou X., Chang D., Huang Q., Li L. (2023). Puerarin Inhibited Oxidative Stress and Alleviated Cerebral Ischemia-Reperfusion Injury through Pi3k/Akt/Nrf2 Signaling Pathway. Front. Pharmacol..

[123] Yen P.T., Huang S.E., Hsu J.H., Kuo C.H., Chao Y.Y., Wang L.S., Yeh J.L. (2023). Anti-Inflammatory and Anti-Oxidative Effects of Puerarin in Postmenopausal Cardioprotection: Roles of Akt and Heme Oxygenase-1. Am. J. Chin. Med..

[124] Trinh P.T.N., Truc N.C., Danh T.T., Trang N.T.T., Le Hang D.T., Vi L.N.T., Hung Q.T., Dung L.T. (2024). A Study on the Antioxidant, Anti-Inflammatory, and Xanthine Oxidase Inhibitory Activity of the *Artemisia vulgaris* L. Extract and Its Fractions. J. Ethnopharmacol..

[125] Rauf A., Imran M., Abu-Izneid T., Iahtisham Ul H., Patel S., Pan X., Naz S., Sanches Silva A., Saeed F., Rasul Suleria H.A. (2019). Proanthocyanidins: A Comprehensive Review. Biomed. Pharmacother..

[126] Oliveira G.A., Ferraz E.R., Souza A.O., Lourenço R.A., Oliveira D.P., Dorta D.J. (2012). Evaluation of the Mutagenic Activity of Chrysin, a Flavonoid Inhibitor of the Aromatization Process. J. Toxicol. Environ. Health A.

[127] Chen Z., Ding W., Yang X., Lu T., Liu Y. (2024). Isoliquiritigenin, a Potential Therapeutic Agent for Treatment of Inflammation-Associated Diseases. J. Ethnopharmacol..

[128] Qiu M., Ma K., Zhang J., Zhao Z., Wang S., Wang Q., Xu H. (2024). Isoliquiritigenin as a Modulator of the Nrf2 Signaling Pathway: Potential Therapeutic Implications. Front. Pharmacol..

[129] Wilmsen P.K., Spada D.S., Salvador M. (2005). Antioxidant Activity of the Flavonoid Hesperidin in Chemical and Biological Systems. J. Agric. Food Chem..

[130] Selvaraj P., Pugalendi K.V. (2010). Hesperidin, a Flavanone Glycoside, on Lipid Peroxidation and Antioxidant Status in Experimental Myocardial Ischemic Rats. Redox Rep..

[131] He J., Liao J.H. (2025). Potential Role of Hesperidin in Improving Experimental Pulmonary Arterial Hypertension in Rats Via Modulation of the Nf-Κb Pathway. Chem. Biol. Drug Des..

[132] Erdem I., Aktas S., Ogut S. (2024). Neohesperidin Dihydrochalcone Ameliorates Experimental Colitis Via Anti-Inflammatory, Antioxidative, and Antiapoptosis Effects. J. Agric. Food Chem..

[133] Autieri M.V., Carbone C.M. (2001). Overexpression of Allograft Inflammatory Factor-1 Promotes Proliferation of Vascular Smooth Muscle Cells by Cell Cycle Deregulation. Arterioscler. Thromb. Vasc. Biol..

[134] Correale M., Tricarico L., Bevere E.M.L., Chirivì F., Croella F., Severino P., Mercurio V., Magrì D., Dini F., Licordari R. (2024). Circulating Biomarkers in Pulmonary Arterial Hypertension: An Update. Biomolecules.

[135] Boucly A., Tu L., Guignabert C., Rhodes C., De Groote P., Prévot G., Bergot E., Bourdin A., Beurnier A., Roche A. (2023). Cytokines as Prognostic Biomarkers in Pulmonary Arterial Hypertension. Eur. Respir. J..

[136] Tobal R., Potjewijd J., de Vries F., van Doorn D.P.C., Jaminon A., Bittner R., Akbulut C., van Empel V., Heeringa P., Damoiseaux J. (2024). Dephosphorylated Uncarboxylated Matrix-Gla-Protein as Candidate Biomarker for Immune-Mediated Vascular Remodeling and Prognosis in Pulmonary Hypertension. Sci. Rep..

[137] Yan Q., Li P., Liu S., Sun Y., Chen C., Long J., Lin Y., Liang J., Wang H., Zhang L. (2024). Dihydromyricetin Treats Pulmonary Hypertension by Modulating Cklf1/Ccr5 Axis-Induced Pulmonary Vascular Cell Pyroptosis. Biomed. Pharmacother..

[138] Li X.Q., Wang H.M., Yang C.G., Zhang X.H., Han D.D., Wang H.L. (2011). Fluoxetine Inhibited Extracellular Matrix of Pulmonary Artery and Inflammation of Lungs in Monocrotaline-Treated Rats. Acta Pharmacol. Sin..

[139] You S., Qian J., Wu G., Qian Y., Wang Z., Chen T., Wang J., Huang W., Liang G. (2019). Schizandrin B Attenuates Angiotensin Ii Induced Endothelial to Mesenchymal Transition in Vascular Endothelium by Suppressing Nf-Κb Activation. Phytomedicine.

[140] Savale L., Tu L., Normand C., Boucly A., Sitbon O., Montani D., Olsson K.M., Park D.H., Fuge J., Kamp J.C. (2024). Effect of Sotatercept on Circulating Proteomics in Pulmonary Arterial Hypertension. Eur. Respir. J..

[141] Bunbupha S., Prachaney P., Kukongviriyapan U., Kukongviriyapan V., Welbat J.U., Pakdeechote P. (2015). Asiatic Acid Alleviates Cardiovascular Remodelling in Rats with L-Name-Induced Hypertension. Clin. Exp. Pharmacol. Physiol..

[142] Ndisang J.F., Chibbar R., Lane N. (2014). Heme Oxygenase Suppresses Markers of Heart Failure and Ameliorates Cardiomyopathy in L-Name-Induced Hypertension. Eur. J. Pharmacol..

[143] Crosswhite P., Sun Z. (2024). Tnfα Induces DNA and Histone Hypomethylation and Pulmonary Artery Smooth Muscle Cell Proliferation Partly Via Excessive Superoxide Formation. Antioxidants.

[144] Kang K., Xiang J., Zhang X., Xie Y., Zhou M., Zeng L., Zhuang J., Kuang J., Lin Y., Hu B. (2024). N6-Methyladenosine Modification of Klf2 May Contribute to Endothelial-to-Mesenchymal Transition in Pulmonary Hypertension. Cell Mol. Biol. Lett..

[145] Zelko I.N., Zhu J., Ritzenthaler J.D., Roman J. (2016). Pulmonary Hypertension and Vascular Remodeling in Mice Exposed to Crystalline Silica. Respir. Res..

[146] Luan Y., Chao S., Ju Z.Y., Wang J., Xue X., Qi T.G., Cheng G.H., Kong F. (2015). Therapeutic Effects of Baicalin on Monocrotaline-Induced Pulmonary Arterial Hypertension by Inhibiting Inflammatory Response. Int. Immunopharmacol..

[147] Teng C., Li B., Lin C., Xing X., Huang F., Yang Y., Li Y., Azevedo H.S., He W. (2022). Targeted Delivery of Baicalein-P53 Complex to Smooth Muscle Cells Reverses Pulmonary Hypertension. J. Control. Release.

[148] Lee J.J., Lee J.H., Yim N.H., Han J.H., Ma J.Y. (2017). Application of Galangin, an Active Component of Alpinia Officinarum Hance (Zingiberaceae), for Use in Drug-Eluting Stents. Sci. Rep..

[149] Collins T., Read M.A., Neish A.S., Whitley M.Z., Thanos D., Maniatis T. (1995). Transcriptional Regulation of Endothelial Cell Adhesion Molecules: Nf-Kappa B and Cytokine-Inducible Enhancers. FASEB J..

[150] Preiss D.J., Sattar N. (2007). Vascular Cell Adhesion Molecule-1: A Viable Therapeutic Target for Atherosclerosis?. Int. J. Clin. Pract..

[151] Zhang Z., Chen H., Chen L., Liang W., Hu T., Sun N., Zhao Y., Wei X. (2025). Blood Pressure and the Risk of Diabetes: A Longitudinal Observational Study Based on Chinese Individuals. J. Diabetes Investig..

[152] Pal H.C., Pearlman R.L., Afaq F. (2016). Fisetin and Its Role in Chronic Diseases. Adv. Exp. Med. Biol..

[153] Sim H., Choo S., Kim J., Baek M.C., Bae J.S. (2020). Fisetin Suppresses Pulmonary Inflammatory Responses through Heme Oxygenase-1 Mediated Downregulation of Inducible Nitric Oxide Synthase. J. Med. Food.

[154] Shi Y., Massagué J. (2003). Mechanisms of Tgf-Beta Signaling from Cell Membrane to the Nucleus. Cell.

[155] Jin Z., Sato Y., Kawashima M., Kanehisa M. (2023). Kegg Tools for Classification and Analysis of Viral Proteins. Protein Sci..

[156] Massagué J. (2000). How Cells Read Tgf-Beta Signals. Nat. Rev. Mol. Cell Biol..

[157] Derynck R., Zhang Y.E. (2003). Smad-Dependent and Smad-Independent Pathways in Tgf-Beta Family Signalling. Nature.

[158] Miguel-Carrasco J.L., Mate A., Monserrat M.T., Arias J.L., Aramburu O., Vázquez C.M. (2008). The role of inflammatory markers in the cardioprotective effect of L-carnitine in L-NAME-induced hypertension. Am. J. Hypertens..

[159] Nyby M.D., Abedi K., Smutko V., Eslami P., Tuck M.L. (2007). Vascular Angiotensin Type 1 Receptor Expression Is Associated with Vascular Dysfunction, Oxidative Stress and Inflammation in Fructose-Fed Rats. Hypertens. Res..

[160] Morris L., Graham C.F., Gordon S. (1991). Macrophages in Haemopoietic and Other Tissues of the Developing Mouse Detected by the Monoclonal Antibody F4/80. Development.

[161] Bertrand J.Y., Jalil A., Klaine M., Jung S., Cumano A., Godin I. (2005). Three Pathways to Mature Macrophages in the Early Mouse Yolk Sac. Blood.

[162] Mossadegh-Keller N., Gentek R., Gimenez G., Bigot S., Mailfert S., Sieweke M.H. (2017). Developmental Origin and Maintenance of Distinct Testicular Macrophage Populations. J. Exp. Med..

[163] Kottmann R.M., Kulkarni A.A., Smolnycki K.A., Lyda E., Dahanayake T., Salibi R., Honnons S., Jones C., Isern N.G., Hu J.Z. (2012). Lactic Acid Is Elevated in Idiopathic Pulmonary Fibrosis and Induces Myofibroblast Differentiation Via Ph-Dependent Activation of Transforming Growth Factor-Β. Am. J. Respir. Crit. Care Med..

[164] Liu C., Jiang X.M., Zhang J., Li B., Li J., Xie D.J., Hu Z.Y. (2016). Pulmonary Artery Denervation Improves Pulmonary Arterial Hypertension Induced Right Ventricular Dysfunction by Modulating the Local Renin-Angiotensin-Aldosterone System. BMC Cardiovasc. Disord..

[165] Abdul-Hafez A., Shu R., Uhal B.D. (2009). Jund and Hif-1alpha Mediate Transcriptional Activation of Angiotensinogen by Tgf-Beta1 in Human Lung Fibroblasts. FASEB J..

[166] Huang S., Chen P., Shui X., He Y., Wang H., Zheng J., Zhang L., Li J., Xue Y., Chen C. (2014). Baicalin Attenuates Transforming Growth Factor-Β1-Induced Human Pulmonary Artery Smooth Muscle Cell Proliferation and Phenotypic Switch by Inhibiting Hypoxia Inducible Factor-1α and Aryl Hydrocarbon Receptor Expression. J. Pharm. Pharmacol..

[167] Li L., Zhang X., Li X., Lv C., Yu H., Xu M., Zhang M., Fu Y., Meng H., Zhou J. (2016). Tgf-Β1 Inhibits the Apoptosis of Pulmonary Arterial Smooth Muscle Cells and Contributes to Pulmonary Vascular Medial Thickening Via the Pi3k/Akt Pathway. Mol. Med. Rep..

[168] Cao S., Zheng B., Chen T., Chang X., Yin B., Huang Z., Shuai P., Han L. (2018). Semen Brassicae Ameliorates Hepatic Fibrosis by Regulating Transforming Growth Factor-Β1/Smad, Nuclear Factor-Κb, and Akt Signaling Pathways in Rats. Drug Des. Devel. Ther..

[169] Bowen T., Jenkins R.H., Fraser D.J. (2013). Micrornas, Transforming Growth Factor Beta-1, and Tissue Fibrosis. J. Pathol..

[170] Xu F., Liu C., Zhou D., Zhang L. (2016). Tgf-Β/Smad Pathway and Its Regulation in Hepatic Fibrosis. J. Histochem. Cytochem..

[171] Xie J., Hu D., Niu L., Qu S., Wang S., Liu S. (2012). Mesenchymal Stem Cells Attenuate Vascular Remodeling in Monocrotaline-Induced Pulmonary Hypertension Rats. J. Huazhong Univ. Sci. Technol. Med. Sci..

[172] Gao W., Shao R., Zhang X., Liu D., Liu Y., Fa X. (2017). Up-Regulation of Caveolin-1 by Dj-1 Attenuates Rat Pulmonary Arterial Hypertension by Inhibiting Tgfβ/Smad Signaling Pathway. Exp. Cell Res..

[173] Shukla S., Gupta S. (2007). Apigenin-Induced Cell Cycle Arrest Is Mediated by Modulation of Mapk, Pi3k-Akt, and Loss of Cyclin D1 Associated Retinoblastoma Dephosphorylation in Human Prostate Cancer Cells. Cell Cycle.

[174] Czyz J., Madeja Z., Irmer U., Korohoda W., Hülser D.F. (2005). Flavonoid Apigenin Inhibits Motility and Invasiveness of Carcinoma Cells in Vitro. Int. J. Cancer.

[175] Chen L., Zhao W. (2016). Apigenin Protects against Bleomycin-Induced Lung Fibrosis in Rats. Exp. Ther. Med..

[176] Wilson D.W., Segall H.J., Pan L.C., Dunston S.K. (1989). Progressive Inflammatory and Structural Changes in the Pulmonary Vasculature of Monocrotaline-Treated Rats. Microvasc. Res..

[177] Golembeski S.M., West J., Tada Y., Fagan K.A. (2005). Interleukin-6 Causes Mild Pulmonary Hypertension and Augments Hypoxia-Induced Pulmonary Hypertension in Mice. Chest.

[178] Woo H.J., Kang H.K., Nguyen T.T., Kim G.E., Kim Y.M., Park J.S., Kim D., Cha J., Moon Y.H., Nam S.H. (2012). Synthesis and Characterization of Ampelopsin Glucosides Using Dextransucrase from Leuconostoc Mesenteroides B-1299cb4: Glucosylation Enhancing Physicochemical Properties. Enzyme Microb. Technol..

[179] Hou X., Tong Q., Wang W., Xiong W., Shi C., Fang J. (2015). Dihydromyricetin Protects Endothelial Cells from Hydrogen Peroxide-Induced Oxidative Stress Damage by Regulating Mitochondrial Pathways. Life Sci..

[180] Wang R.N., Green J., Wang Z., Deng Y., Qiao M., Peabody M., Zhang Q., Ye J., Yan Z., Denduluri S. (2014). Bone Morphogenetic Protein (Bmp) Signaling in Development and Human Diseases. Genes Dis..

[181] Lau E.M.T., Giannoulatou E., Celermajer D.S., Humbert M. (2017). Epidemiology and Treatment of Pulmonary Arterial Hypertension. Nat. Rev. Cardiol..

[182] Atkinson C., Stewart S., Upton P.D., Machado R., Thomson J.R., Trembath R.C., Morrell N.W. (2002). Primary Pulmonary Hypertension Is Associated with Reduced Pulmonary Vascular Expression of Type Ii Bone Morphogenetic Protein Receptor. Circulation.

[183] Lane K.B., Machado R.D., Pauciulo M.W., Thomson J.R., Phillips J.A., Loyd J.E., Nichols W.C., Trembath R.C. (2000). Heterozygous Germline Mutations in Bmpr2, Encoding a Tgf-Beta Receptor, Cause Familial Primary Pulmonary Hypertension. Nat. Genet..

[184] Hansmann G., Calvier L., Risbano M.G., Chan S.Y. (2020). Activation of the Metabolic Master Regulator Pparγ: A Potential Pioneering Therapy for Pulmonary Arterial Hypertension. Am. J. Respir. Cell Mol. Biol..

[185] Shimizu T., Higashijima Y., Kanki Y., Nakaki R., Kawamura T., Urade Y., Wada Y. (2021). Perk Inhibition Attenuates Vascular Remodeling in Pulmonary Arterial Hypertension Caused by Bmpr2 Mutation. Sci. Signal.

[186] Frump A., Prewitt A., de Caestecker M.P. (2018). Bmpr2 Mutations and Endothelial Dysfunction in Pulmonary Arterial Hypertension (2017 Grover Conference Series). Pulm. Circ..

[187] Tielemans B., Delcroix M., Belge C., Quarck R. (2019). Tgfβ and Bmprii Signalling Pathways in the Pathogenesis of Pulmonary Arterial Hypertension. Drug Discov. Today.

[188] Abdalla S., Zarga M.A., Afifi F., al-Khalil S., Mahasneh A., Sabri S. (1989). Effects of 3,3′-Di-O-Methylquercetin on Guinea-Pig Isolated Smooth Muscle. J. Pharm. Pharmacol..

[189] Luo S., Kan J., Zhang J., Ye P., Wang D., Jiang X., Li M., Zhu L., Gu Y. (2021). Bioactive Compounds from Coptidis Rhizoma Alleviate Pulmonary Arterial Hypertension by Inhibiting Pulmonary Artery Smooth Muscle Cells’ Proliferation and Migration. J. Cardiovasc. Pharmacol..

[190] Goncharov D.A., Kudryashova T.V., Ziai H., Ihida-Stansbury K., DeLisser H., Krymskaya V.P., Tuder R.M., Kawut S.M., Goncharova E.A. (2014). Mammalian Target of Rapamycin Complex 2 (Mtorc2) Coordinates Pulmonary Artery Smooth Muscle Cell Metabolism, Proliferation, and Survival in Pulmonary Arterial Hypertension. Circulation.

[191] Goncharova E.A. (2013). Mtor and Vascular Remodeling in Lung Diseases: Current Challenges and Therapeutic Prospects. FASEB J..

[192] Houssaini A., Abid S., Mouraret N., Wan F., Rideau D., Saker M., Marcos E., Tissot C.M., Dubois-Randé J.L., Amsellem V. (2013). Rapamycin Reverses Pulmonary Artery Smooth Muscle Cell Proliferation in Pulmonary Hypertension. Am. J. Respir. Cell Mol. Biol..

[193] Walker E.H., Pacold M.E., Perisic O., Stephens L., Hawkins P.T., Wymann M.P., Williams R.L. (2000). Structural Determinants of Phosphoinositide 3-Kinase Inhibition by Wortmannin, Ly294002, Quercetin, Myricetin, and Staurosporine. Mol. Cell.

[194] Morales-Cano D., Menendez C., Moreno E., Moral-Sanz J., Barreira B., Galindo P., Pandolfi R., Jimenez R., Moreno L., Cogolludo A. (2014). The Flavonoid Quercetin Reverses Pulmonary Hypertension in Rats. PLoS ONE.

[195] Sutliff R.L., Kang B.Y., Hart C.M. (2010). Ppargamma as a Potential Therapeutic Target in Pulmonary Hypertension. Ther. Adv. Respir. Dis..

[196] Peng X., Chen R., Wu Y., Huang B., Tang C., Chen J., Wang Q., Wu Q., Yang J., Qiu H. (2015). Pparγ-Pi3k/Akt-No Signal Pathway Is Involved in Cardiomyocyte Hypertrophy Induced by High Glucose and Insulin. J. Diabetes Complicat..

[197] Yasuda S., Kobayashi H., Iwasa M., Kawamura I., Sumi S., Narentuoya B., Yamaki T., Ushikoshi H., Nishigaki K., Nagashima K. (2009). Antidiabetic Drug Pioglitazone Protects the Heart Via Activation of Ppar-Gamma Receptors, Pi3-Kinase, Akt, and Enos Pathway in a Rabbit Model of Myocardial Infarction. Am. J. Physiol. Heart Circ. Physiol..

[198] Rabinovitch M. (2010). Ppargamma and the Pathobiology of Pulmonary Arterial Hypertension. Adv. Exp. Med. Biol..

[199] Zhang Y., Wang Y., Yang K., Tian L., Fu X., Wang Y., Sun Y., Jiang Q., Lu W., Wang J. (2014). Bmp4 Increases the Expression of Trpc and Basal [Ca^2+^]I Via the P38mapk and Erk1/2 Pathways Independent of Bmprii in Pasmcs. PLoS ONE.

[200] Wilson J.L., Yu J., Taylor L., Polgar P. (2015). Hyperplastic Growth of Pulmonary Artery Smooth Muscle Cells from Subjects with Pulmonary Arterial Hypertension Is Activated through Jnk and P38 Mapk. PLoS ONE.

[201] Dang J., Zhang Z., Fu J., Sun L., Shi Y., Wang L., Tao W., Cheng D., Wang X., Mi Z. (2025). Regulating Inflammation Microenvironment and Tenogenic Differentiation as Sequential Therapy Promotes Tendon Healing in Diabetic Rats. J. Orthop. Transl..

[202] Ottolenghi S., Zulueta A., Caretti A. (2020). Iron and Sphingolipids as Common Players of (Mal)Adaptation to Hypoxia in Pulmonary Diseases. Int. J. Mol. Sci..

[203] Kong C.C., Dai A.G. (2006). Expression of Mitogen-Actived Protein Kinase, Phosphatidylinositol 3-Kinase and Hypoxia-Inducible Factor-1α in Pulmonary Arteries of Patients with Chronic Obstructive Pulmonary Disease. Chin. J. Tuberc. Respir. Dis..

[204] Deng Z.H., Chen Y.X., Xue G., Yang J.Y., Wei X.Y., Zhang G.X., Qian J.X. (2024). Mesenchymal Stem Cell-Derived Exosomes Ameliorate Hypoxic Pulmonary Hypertension by Inhibiting the Hsp90aa1/Erk/Perk Pathway. Biochem. Pharmacol..

[205] Tsai H.H., Chen I.J., Lo Y.C. (2008). Effects of San-Huang-Xie-Xin-Tang on U46619-Induced Increase in Pulmonary Arterial Blood Pressure. J. Ethnopharmacol..

[206] Barnes H., Brown Z., Burns A., Williams T. (2019). Phosphodiesterase 5 Inhibitors for Pulmonary Hypertension. Cochrane Database Syst. Rev..

[207] Won K.J., Lee K.P., Baek S., Cui L., Kweon M.H., Jung S.H., Ryu Y.K., Hong J.M., Cho E.A., Shin H.S. (2017). Desalted Salicornia Europaea Extract Attenuated Vascular Neointima Formation by Inhibiting the Mapk Pathway-Mediated Migration and Proliferation in Vascular Smooth Muscle Cells. Biomed. Pharmacother..

[208] Weerackody R.P., Welsh D.J., Wadsworth R.M., Peacock A.J. (2009). Inhibition of P38 Mapk Reverses Hypoxia-Induced Pulmonary Artery Endothelial Dysfunction. Am. J. Physiol. Heart Circ. Physiol..

[209] Rakotomalala G., Agard C., Tonnerre P., Tesse A., Derbré S., Michalet S., Hamzaoui J., Rio M., Cario-Toumaniantz C., Richomme P. (2013). Extract from Mimosa Pigra Attenuates Chronic Experimental Pulmonary Hypertension. J. Ethnopharmacol..

[210] Krenn L., Paper D.H. (2009). Inhibition of Angiogenesis and Inflammation by an Extract of Red Clover (*Trifolium Pratense* L.). Phytomedicine.

[211] Lammers S.R., Kao P.H., Qi H.J., Hunter K., Lanning C., Albietz J., Hofmeister S., Mecham R., Stenmark K.R., Shandas R. (2008). Changes in the Structure-Function Relationship of Elastin and Its Impact on the Proximal Pulmonary Arterial Mechanics of Hypertensive Calves. Am. J. Physiol. Heart Circ. Physiol..

[212] Negrão R., Costa R., Duarte D., Taveira Gomes T., Mendanha M., Moura L., Vasques L., Azevedo I., Soares R. (2010). Angiogenesis and Inflammation Signaling Are Targets of Beer Polyphenols on Vascular Cells. J. Cell Biochem..

[213] Silva A.F., Faria-Costa G., Sousa-Nunes F., Santos M.F., Ferreira-Pinto M.J., Duarte D., Rodrigues I., Tiago Guimarães J., Leite-Moreira A., Moreira-Gonçalves D. (2019). Anti-Remodeling Effects of Xanthohumol-Fortified Beer in Pulmonary Arterial Hypertension Mediated by Erk and Akt Inhibition. Nutrients.

[214] Nguyen T., Yang C.S., Pickett C.B. (2004). The Pathways and Molecular Mechanisms Regulating Nrf2 Activation in Response to Chemical Stress. Free Radic. Biol. Med..

[215] Nguyen T., Sherratt P.J., Huang H.C., Yang C.S., Pickett C.B. (2003). Increased protein stability as a mechanism that enhances Nrf2-mediated transcriptional activation of the antioxidant response element. Degradation of Nrf2 by the 26 S proteasome. J. Biol. Chem..

[216] Wang J., Huang X., Zhang K., Mao X., Ding X., Zeng Q., Bai S., Xuan Y., Peng H. (2017). Vanadate Oxidative and Apoptotic Effects Are Mediated by the Mapk-Nrf2 Pathway in Layer Oviduct Magnum Epithelial Cells. Metallomics.

[217] Aladaileh S.H., Hussein O.E., Abukhalil M.H., Saghir S.A.M., Bin-Jumah M., Alfwuaires M.A., Germoush M.O., Almaiman A.A., Mahmoud A.M. (2019). Formononetin Upregulates Nrf2/Ho-1 Signaling and Prevents Oxidative Stress, Inflammation, and Kidney Injury in Methotrexate-Induced Rats. Antioxidants.

[218] Ko W., Lee H., Kim N., Jo H.G., Woo E.R., Lee K., Han Y.S., Park S.R., Ahn G., Cheong S.H. (2021). The Anti-Oxidative and Anti-Neuroinflammatory Effects of Sargassum Horneri by Heme Oxygenase-1 Induction in Bv2 and Ht22 Cells. Antioxidants.

[219] Liu Q., Ci X., Wen Z., Peng L. (2018). Diosmetin Alleviates Lipopolysaccharide-Induced Acute Lung Injury through Activating the Nrf2 Pathway and Inhibiting the Nlrp3 Inflammasome. Biomol. Ther..

[220] Choi S.Y., Ko H.C., Ko S.Y., Hwang J.H., Park J.G., Kang S.H., Han S.H., Yun S.H., Kim S.J. (2007). Correlation between Flavonoid Content and the No Production Inhibitory Activity of Peel Extracts from Various Citrus Fruits. Biol. Pharm. Bull..

[221] Murakami A., Nakamura Y., Ohto Y., Yano M., Koshiba T., Koshimizu K., Tokuda H., Nishino H., Ohigashi H. (2000). Suppressive Effects of Citrus Fruits on Free Radical Generation and Nobiletin, an Anti-Inflammatory Polymethoxyflavonoid. Biofactors.

[222] Wu X., Song M., Rakariyatham K., Zheng J., Guo S., Tang Z., Zhou S., Xiao H. (2015). Anti-Inflammatory Effects of 4′-Demethylnobiletin, a Major Metabolite of Nobiletin. J. Funct. Foods.

[223] Farkas D., Alhussaini A.A., Kraskauskas D., Kraskauskiene V., Cool C.D., Nicolls M.R., Natarajan R., Farkas L. (2014). Nuclear Factor Κb Inhibition Reduces Lung Vascular Lumen Obliteration in Severe Pulmonary Hypertension in Rats. Am. J. Respir. Cell Mol. Biol..

[224] Zhang N., Qiu Q., Chen Y., Sun Z., Lu G., Wang L., Kang P., Wang H. (2023). Quercetin Improves Pulmonary Arterial Hypertension in Rats by Regulating the Hmgb1/Rage/Nf-Κb Pathway. J. South. Med. Univ..

[225] Li Y., Yang L., Dong L., Yang Z.W., Zhang J., Zhang S.L., Niu M.J., Xia J.W., Gong Y., Zhu N. (2019). Crosstalk between the Akt/Mtorc1 and Nf-Κb Signaling Pathways Promotes Hypoxia-Induced Pulmonary Hypertension by Increasing Dpp4 Expression in Pasmcs. Acta Pharmacol. Sin..

[226] Xia J., Yang L., Dong L., Niu M., Zhang S., Yang Z., Wumaier G., Li Y., Wei X., Gong Y. (2018). Cefminox, a Dual Agonist of Prostacyclin Receptor and Peroxisome Proliferator-Activated Receptor-Gamma Identified by Virtual Screening, Has Therapeutic Efficacy against Hypoxia-Induced Pulmonary Hypertension in Rats. Front. Pharmacol..

[227] Parameswaran N., Patial S. (2010). Tumor Necrosis Factor-α Signaling in Macrophages. Crit. Rev. Eukaryot. Gene Expr..

[228] Milstone D.S., Ilyama M., Chen M., O’Donnell P., Davis V.M., Plutzky J., Brown J.D., Haldar S.M., Siu A., Lau A.C. (2015). Differential Role of an Nf-Κb Transcriptional Response Element in Endothelial Versus Intimal Cell Vcam-1 Expression. Circ. Res..

[229] Zhou M.S., Hernandez Schulman I., Pagano P.J., Jaimes E.A., Raij L. (2006). Reduced Nad(P)H Oxidase in Low Renin Hypertension: Link among Angiotensin Ii, Atherogenesis, and Blood Pressure. Hypertension.

[230] Li X., Lin Y., Zhou H., Li Y., Wang A., Wang H., Zhou M.S. (2017). Puerarin Protects against Endothelial Dysfunction and End-Organ Damage in Ang Ii-Induced Hypertension. Clin. Exp. Hypertens..

[231] Tan C., Wang A., Liu C., Li Y., Shi Y., Zhou M.S. (2017). Puerarin Improves Vascular Insulin Resistance and Cardiovascular Remodeling in Salt-Sensitive Hypertension. Am. J. Chin. Med..

[232] Garat C.V., Crossno J.T., Sullivan T.M., Reusch J.E., Klemm D.J. (2013). Inhibition of Phosphatidylinositol 3-Kinase/Akt Signaling Attenuates Hypoxia-Induced Pulmonary Artery Remodeling and Suppresses Creb Depletion in Arterial Smooth Muscle Cells. J. Cardiovasc. Pharmacol..

[233] Wang J., Bian Y., Cheng Y., Sun R., Li G. (2020). Effect of Lemon Peel Flavonoids on Uvb-Induced Skin Damage in Mice. RSC Adv..

[234] Lam K.H., Alex D., Lam I.K., Tsui S.K., Yang Z.F., Lee S.M. (2011). Nobiletin, a Polymethoxylated Flavonoid from Citrus, Shows Anti-Angiogenic Activity in a Zebrafish in Vivo Model and Huvec in Vitro Model. J. Cell Biochem..

[235] Saikumar P., Dong Z., Mikhailov V., Denton M., Weinberg J.M., Venkatachalam M.A. (1999). Apoptosis: Definition, Mechanisms, and Relevance to Disease. Am. J. Med..

[236] Nicholson K.M., Anderson N.G. (2002). The Protein Kinase B/Akt Signalling Pathway in Human Malignancy. Cell Signal.

[237] Manning B.D., Cantley L.C. (2007). Akt/Pkb Signaling: Navigating Downstream. Cell.

[238] Huertas A., Guignabert C., Barberà J.A., Bärtsch P., Bhattacharya J., Bhattacharya S., Bonsignore M.R., Dewachter L., Dinh-Xuan A.T., Dorfmüller P. (2018). Pulmonary Vascular Endothelium: The Orchestra Conductor in Respiratory Diseases: Highlights from Basic Research to Therapy. Eur. Respir. J..

[239] Christou H., Khalil R.A. (2022). Mechanisms of Pulmonary Vascular Dysfunction in Pulmonary Hypertension and Implications for Novel Therapies. Am. J. Physiol. Heart Circ. Physiol..

[240] Chester A.H., Yacoub M.H., Moncada S. (2017). Nitric Oxide and Pulmonary Arterial Hypertension. Glob. Cardiol. Sci. Pract..

[241] Feriel B., Alessandra C., Deborah G.J., Corinne N., Raphaël T., Mina O., Ali A., Jean-Baptiste M., Guillaume F., Julien G. (2024). Exploring the Endothelin-1 Pathway in Chronic Thromboembolic Pulmonary Hypertension Microvasculopathy. Sci. Rep..

[242] Dai J., Chen H., Fang J., Wu S., Jia Z. (2025). Vascular Remodeling: The Multicellular Mechanisms of Pulmonary Hypertension. Int. J. Mol. Sci..

[243] Yan L.P., Chan S.W., Chan A.S., Chen S.L., Ma X.J., Xu H.X. (2006). Puerarin Decreases Serum Total Cholesterol and Enhances Thoracic Aorta Endothelial Nitric Oxide Synthase Expression in Diet-Induced Hypercholesterolemic Rats. Life Sci..

[244] Khoo N.K., White C.R., Pozzo-Miller L., Zhou F., Constance C., Inoue T., Patel R.P., Parks D.A. (2010). Dietary Flavonoid Quercetin Stimulates Vasorelaxation in Aortic Vessels. Free Radic. Biol. Med..

[245] Lai Y.J., Hsu H.H., Chang G.J., Lin S.H., Chen W.J., Huang C.C., Pang J.S. (2017). Prostaglandin E1 Attenuates Pulmonary Artery Remodeling by Activating Phosphorylation of Creb and the Pten Signaling Pathway. Sci. Rep..

[246] Cheng M., Li X., Guo Z., Cui X., Li H., Jin C., Zhang X., Guan X. (2013). Puerarin Accelerates Re-Endothelialization in a Carotid Arterial Injury Model: Impact on Vasodilator Concentration and Vascular Cell Functions. J. Cardiovasc. Pharmacol..

[247] Wei X., Zhu X., Hu N., Zhang X., Sun T., Xu J., Bian X. (2015). Baicalin Attenuates Angiotensin Ii-Induced Endothelial Dysfunction. Biochem. Biophys. Res. Commun..

[248] Luo S., Li S., Zhu L., Fang S.H., Chen J.L., Xu Q.Q., Li H.Y., Luo N.C., Yang C., Luo D. (2017). Effect of Baicalin on Oxygen-Glucose Deprivation-Induced Endothelial Cell Damage. Neuroreport.

[249] Wang L., Li Y., Lin S., Pu Z., Li H., Tang Z. (2018). Protective Effects of Baicalin on Experimental Myocardial Infarction in Rats. Braz. J. Cardiovasc. Surg..

[250] Zhang S., Li X., Yao L., Guo L., Jiang Y., Jin H. (2018). Effect of Isoliquiritigenin on Hypoxia-Induced Pulmonary Artery Remodeling in Rats. Acta Anat. Sin..

[251] Naz S., Imran M., Rauf A., Orhan I.E., Shariati M.A., Iahtisham Ul H., IqraYasmin, Shahbaz M., Qaisrani T.B., Shah Z.A. (2019). Chrysin: Pharmacological and Therapeutic Properties. Life Sci..

[252] Amato C. (1994). Advantage of a Micronized Flavonoidic Fraction (Daflon 500 Mg) in Comparison with a Nonmicronized Diosmin. Angiology.

[253] Carpentier P., Karetova D., Gutierrez L.R., Maggioli A. (2018). 19th Meeting of the Europeanvenous Forum: Athens, Greece. Phlebology.

[254] (2017). Abstracts from the 38th Annual Scientific Meeting of the Hbprca. Hypertension.

[255] Grassi D., Desideri G., De Feo M., Fellini E., Mai F., Dante A., Di Agostino S., Di Giosia P., Patrizi F., Martella L. (2014). XXXI National Congress of the Italian Society of Hypertension (Siia) Selected Abstracts. High Blood Press. Cardiovasc. Prev..

[256] Engler M.B., Engler M.M., Chen C.Y., Malloy M.J., Browne A., Chiu E.Y., Kwak H.K., Milbury P., Paul S.M., Blumberg J. (2004). Flavonoid-Rich Dark Chocolate Improves Endothelial Function and Increases Plasma Epicatechin Concentrations in Healthy Adults. J. Am. Coll. Nutr..

[257] Alizadeh S.R., Savadkouhi N., Ebrahimzadeh M.A. (2023). Drug Design Strategies That Aim to Improve the Low Solubility and Poor Bioavailability Conundrum in Quercetin Derivatives. Expert. Opin. Drug Discov..

[258] Ribaudo G., Pagano M.A., Pavan V., Redaelli M., Zorzan M., Pezzani R., Mucignat-Caretta C., Vendrame T., Bova S., Zagotto G. (2015). Semi-Synthetic Derivatives of Natural Isoflavones from Maclura Pomifera as a Novel Class of Pde-5a Inhibitors. Fitoterapia.

[259] Pan B., Wu F., Lu S., Lu W., Cao J., Cheng F., Ou M., Chen Y., Zhang F., Wu G. (2025). Luteolin-Loaded Hyaluronidase Nanoparticles with Deep Tissue Penetration Capability for Idiopathic Pulmonary Fibrosis Treatment. Small Methods.

[260] Yu C.Y., Cong Y.J., Wei J.X., Guo B.L., Liu C.Y., Liao Y.H. (2024). Pulmonary Delivery of Icariin-Phospholipid Complex Prolongs Lung Retention and Improves Therapeutic Efficacy in Mice with Acute Lung Injury/Ards. Colloids Surf. B Biointerfaces.

[261] Haneef J., Chadha R. (2019). Implication of Differential Surface Anisotropy on Biopharmaceutical Performance of Polymorphic Forms of Ambrisentan. J. Pharm. Sci..

[262] Galiè N., McLaughlin V.V., Rubin L.J., Simonneau G. (2019). An Overview of the 6th World Symposium on Pulmonary Hypertension. Eur. Respir. J..

